# Whole genome and exome sequencing reference datasets from a multi-center and cross-platform benchmark study

**DOI:** 10.1038/s41597-021-01077-5

**Published:** 2021-11-09

**Authors:** Yongmei Zhao, Li Tai Fang, Tsai-wei Shen, Sulbha Choudhari, Keyur Talsania, Xiongfong Chen, Jyoti Shetty, Yuliya Kriga, Bao Tran, Bin Zhu, Zhong Chen, Wanqiu Chen, Charles Wang, Erich Jaeger, Daoud Meerzaman, Charles Lu, Kenneth Idler, Luyao Ren, Yuanting Zheng, Leming Shi, Virginie Petitjean, Marc Sultan, Tiffany Hung, Eric Peters, Jiri Drabek, Petr Vojta, Roberta Maestro, Daniela Gasparotto, Sulev Kõks, Ene Reimann, Andreas Scherer, Jessica Nordlund, Ulrika Liljedahl, Jonathan Foox, Christopher E. Mason, Chunlin Xiao, Huixiao Hong, Wenming Xiao

**Affiliations:** 1 Advanced Biomedical and Computational Sciences, Biomedical Informatics and Data Science Directorate, Frederick National Laboratory for Cancer Research, Frederick, MD USA; 2 Bioinformatics Research & Early Development, Roche Sequencing Solutions Inc., Belmont, CA USA; 3 Sequencing Facility, Cancer Research Technology Program, Frederick National Laboratory for Cancer Research, Frederick, MD USA; 4 Division of Cancer Epidemiology and Genetics, National Cancer Institute, National Institutes of Health, Bethesda, MD USA; 5 Center for Genomics, School of Medicine, Loma Linda University, Loma Linda, CA USA; 6 Core Applications Group, Product Development, Illumina Inc, Foster City, CA USA; 7 Computational Genomics and Bioinformatics Branch, Center for Biomedical Informatics and Information Technology, National Cancer Institute, National Institutes of Health, Bethesda, MD USA; 8 AbbVie Genomics Research Center, North Chicago, IL USA; 9 State Key Laboratory of Genetic Engineering, School of Life Sciences and Shanghai Cancer Center, Fudan University, Shanghai, China; 10 Biomarker Development, Novartis Institutes for Biomedical Research, Basel, Switzerland; 11 Companion Diagnostics Development, Oncology Biomarker Development, Genentech, South San Francisco, CA USA; 12 IMTM, Faculty of Medicine and Dentistry, Palacky University, Olomouc, Czech Republic; 13 Member of EATRIS ERIC - European Infrastructure for Translational Medicine, Amsterdam, The Netherlands; 14 Centro di Riferimento Oncologico di Aviano (CRO) IRCCS, National Cancer Institute, Unit of Oncogenetics and Functional Oncogenomics, Aviano, Italy; 15 Perron Institute for Neurological and Translational Science, Nedlands, Australia; 16 Centre for Molecular Medicine and Innovative Therapeutics, Murdoch University, Murdoch, Australia; 17 Estonian Genome Centre, Institute of Genomics, University of Tartu, Tartu, Estonia; 18 Institute for Molecular Medicine Finland (FIMM), University of Helsinki, Helsinki, Finland; 19 Department of Medical Sciences, Molecular Precision Medicine and Science for Life Laboratory, Uppsala University, Uppsala, Sweden; 20 Department of Physiology and Biophysics, Weill Cornell Medicine, New York, NY USA; 21 National Center for Biotechnology Information, National Library of Medicine, National Institutes of Health, Bethesda, MD USA; 22 National Center for Toxicological Research, U.S. Food and Drug Administration, FDA, Jefferson, AR USA; 23 The Center for Drug Evaluation and Research, U.S. Food and Drug Administration, FDA, Silver Spring, MD USA

**Keywords:** Data processing, Personalized medicine, Standardization

## Abstract

With the rapid advancement of sequencing technologies, next generation sequencing (NGS) analysis has been widely applied in cancer genomics research. More recently, NGS has been adopted in clinical oncology to advance personalized medicine. Clinical applications of precision oncology require accurate tests that can distinguish tumor-specific mutations from artifacts introduced during NGS processes or data analysis. Therefore, there is an urgent need to develop best practices in cancer mutation detection using NGS and the need for standard reference data sets for systematically measuring accuracy and reproducibility across platforms and methods. Within the SEQC2 consortium context, we established paired tumor-normal reference samples and generated whole-genome (WGS) and whole-exome sequencing (WES) data using sixteen library protocols, seven sequencing platforms at six different centers. We systematically interrogated somatic mutations in the reference samples to identify factors affecting detection reproducibility and accuracy in cancer genomes. These large cross-platform/site WGS and WES datasets using well-characterized reference samples will represent a powerful resource for benchmarking NGS technologies, bioinformatics pipelines, and for the cancer genomics studies.

## Abstract (summary)

**Table:** 

Measurement(s)	Somatic Mutation Analysis
Technology Type(s)	whole genome sequencing • Whole Exome Sequencing
Factor Type(s)	sequencing platform • sample prepration • library preparation • bioinformatics method
Sample Characteristic - Organism	Homo sapiens

Machine-accessible metadata file describing the reported data: 10.6084/m9.figshare.16713655

## Background & Summary

**Fig. 1:**
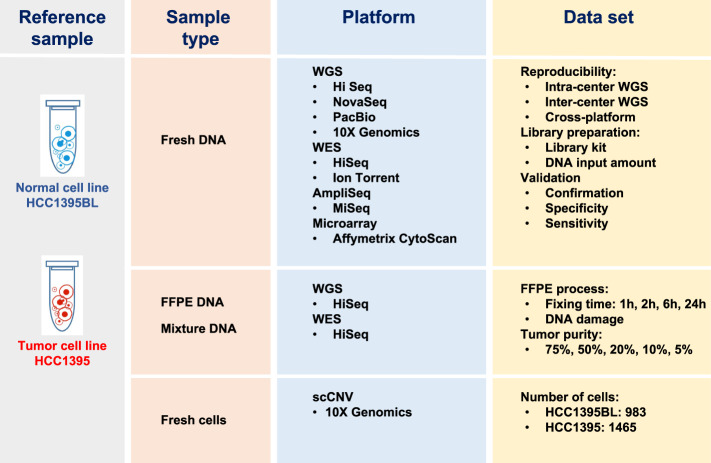
Study design for the experiment. DNA was extracted from either fresh cells or FFPE processed cells. Both fresh DNA and FFPE DNA were profiled on WGS and WES platforms for intra-center, inter-center and cross-platform reproducibility benchmarking. For fresh DNA, six centers performed WGS and WES in parallel following manufacture recommended protocols with limited deviation. Three library preparation protocols (TruSeq-Nano, Nextera Flex, and TruSeq PCR-free,) were used with four different quantities of DNA inputs (1, 10, 100, and 250 ng). DNA from HCC1395 and HCC1395BL was pooled at various ratios to create mixtures of 75%, 50%, 20%, 10%, and 5%. For FFPE samples, each fixation time point (1h, 2 h, 6 h, 24 h) had six blocks that were sequenced at two different centers. All libraries from these experiments were sequenced on the HiSeq series. In addition, nine libraries using the TruSeq PCR-free preparation were run on a NovaSeq for WGS analysis.

The NGS technology has become a powerful tool for precision medicine. More researchers and clinicians are utilizing NGS to identify clinically actionable mutations in cancer patients and to establish targeted therapies for patients based on the patient’s genetic makeup or genetic variants of their tumor^1^, there is a critical need to have a full understanding of the many different variables affecting the NGS analysis output. The rapid growing number of sample processing protocols, library preparation methods, sequencing platforms, and bioinformatics pipelines to detect mutations in cancer genome, presents great technical challenges for the accuracy and reproducibility of utilizing NGS for cancer genome mutation detections. To investigate how these experimental and analytical elements may affect mutation detection accuracy, recently we carried out a comprehensive benchmarking study^2^ using both whole-genome (WGS) and whole-exome sequencing (WES) data sets generated from two well-characterized reference samples: a human breast cancer cell line (HCC1395) and a B lymphocytes cell line (HCC1395BL) derived from the same donor^3^. We generated WGS and WES data using various NGS library preparation protocols, seven NGS platforms (NovaSeq, HiSeq, PacBio, 10X Genomics, Ion Torrent, Miseq, and Affymetrix CytoScan HD) at six centers including Illumina (IL), National Cancer Institute (NC), Novartis (NV), European Infrastructure for Translational Medicine (EA), Fudan University (FD), and Loma Linda University (LL) (Fig. 1).

Figure 1 shows our overall study design. Briefly, DNA was extracted from fresh cells or cell pellets mimicking the formalin-fixed paraffin-embedded (FFPE) process with fixation time of 1, 2, 6, or 24 hours. A small amount of DNA from fresh cells of HCC1395 and HCC1395BL was pooled at various ratios (3:1, 1:1, 1:4, 1:9 and 1:19) to create mixtures. Both fresh DNA and FFPE DNA were profiled on NGS or microarray platforms following manufacturer recommended protocols. To assess the reproducibility of WGS and WES, six sequencing centers performed a total of 12 replicates (3 × 3 + 3) on each platform. In addition, 12 WGS libraries constructed using three different library preparation protocols (TruSeq PCR-free, TruSeq-Nano, and Nextera Flex) in four different quantities of DNA inputs (1, 10, 100, and 250 ng) were sequenced on an Illumina HiSeq 4000, and nine WGS libraries constructed using the TruSeq PCR-free protocol were sequenced on an Illumina NovaSeq. Finally, Affymetrix Cytoscan HD and single-cell sequencing with 10X Genomics platform were performed to uncover the cytogenetics and heterogeneity of two cell lines. Table 1 contains the details of the platform, library protocols and read coverage information.

**Table 1:** Summary of all experiment data including WGS, WES discovery and validation data sets used in the study.

Study Design	Technology	Library Prep (input amount)	Platform	Sequecing	Number of Reads (coverage)	
					HCC1395	HCC1395BL
Discovery	WGS (Fresh DNA)	TruSeq DNA PCR Free (1000 ng)	HiSeq	6 centers (24 libraries)	21 billion (750X)	21 billion (750X)
		TruSeq DNA PCR Free (1000 ng)	NovaSeq	1 center (18 libraries)	13 billion (400X)	13 billion (400X)
	WES (Fresh DNA)	Agilent SureSelect Human All Exon v6 + UTRs	HiSeq	6 centers (24 libraries)	3 billion (2,500X)	3 billion (2,500X)
Validation	WGS Protocol & Input Amount	TruSeq-DNA-Nano (1, 10, 100 ng), Nextera flex (1, 10, 100 ng), TruSeq PCR free (250 ng)	HiSeq	2 center (14 libraries)	9 billion (315X)	9 billion (315X)
	WGS Tumor Content	TruSeq DNA PCR Free (1000 ng)	HiSeq	1 center (21 libraries)	64 billion (Mixture of samples, total 2300X)	
	WGS FFPE	NEBNext Ultra II (200 ng)	HiSeq	2 center (26 libraries)	30 billion (980X)	27 billion (900X)
	WGS 10x Linked-Read	10X Chromium Genome Library Prep v2 (1250 ng)	10X Genomics	5 centers (22 libraries)	20 billion (880X)	20 billion (880X)
	WGS PacBio	Preparing >30 kbp SMRTbell Libraries	PacBio	1 center (2 libraries)	19 million (40X)	22 million (44X)
	WES FFPE	Agilent SureSelect Human All Exon v6 + UTRs	HiSeq	2 centers (17 libraries)	3 billion (2600X)	4 billion (3600X)
	WES	Agilent SureSelect Human All Exon v6 + UTRs	Ion Torrent	1 centers (2 libraries)	67 million (34X)	82 million (47X)
	AmpliSeq	AmpliSeq Targeted Amplicon Panel	MiSeq	1 center (2 libraries)	25 million (2900x)	22 million (3300x)
	Microarray	AffyChip CytoScan	AffyChip CytoScan HD	1 center (2 libraries)	2.1 million probes	
	Single Cell CNV	10x Chromium Single Cell CNV Solution	HiSeq	1 center (2 libraries)	1.5 billion (1465 cells)	1.3 billion (983 cells)

The table columns describe the data sets generated for either Discovery or Validation purpose of the study. For each experiment, the input DNA bio-sample preservation types and sequencing platforms are specified. There are total six sequencing centers for cross-site study. The total number of librarys are listed for HCC1395 and HCC1395BL together while the read coverages for each cell line are listed separately. For the QC statistics for each data set, please reference the Online-Only Tables 1–10 for details.

We first established reference call sets with evidence from 21 replicates of Illumina WGS runs with coverage ranging from 50X to 100X (1150X in total). We split mutation call confidence levels into four categories: HighConf, MedConf, LowConf, and Unclassified^3^. By combining all WGS runs, we were able to further confirm and improve our call set with tumor-normal pairs of 1500X data sets and identified mutations with VAF as low as 1.5%. A subset of reference mutation calls was validated by targeted exome sequencing (WES at 2,500X coverage) using HiSeq, and deep sequencing from AmpliSeq (at 2,000X coverage) using Miseq, and Ion Torrent (at 34X coverage), and long-read WGS by PacBio Sequel (at 40X coverage). In addition, we inferred subclones and heterogeneity of HCC1395 with bulk DNA sequencing. The results were confirmed by single-cell DNA sequencing analysis^3^.

With defined reference call sets, we then systematically interrogated somatic mutations to identify factors affecting detection reproducibility and accuracy. By examining the interactions and effects of NGS platform, library preparation protocol, tumor content, read coverage, and bioinformatics process concomitantly, we observed that each component of the sequencing and analysis process can affect the final outcome. Overall WES and WGS results have high concordance and correlation. WES had a better coverage/cost ratio than WGS. However, sequencing coverage of the WES target regions was not even. In addition, WES showed more batch effects/artifacts due to laboratory processing and thus had larger variation between runs, laboratories, and likely between researchers preparing the libraries. As a result, WES had much larger inter-center variation and was less reproducible than WGS. Biological (library) replicates removed some artifacts due to random events (“Non-Repeatable” calls) and offered much better calling precision than did a single test. Analytical repeats (two bioinformatics pipelines) also increased calling precision at the cost of increased false negatives. We found that biological replicates are more important than bioinformatics replicates in cases where high specificity and sensitivity are needed^1^.

## Methods

Detailed methods were described in our two companion papers^2,3^.

### Cell line culture and DNA extraction

HCC1395; Breast Carcinoma; Human (*Homo sapiens*) cells (expanded from ATCC CRL-2324) were cultured in ATCC-formulated RPMI-1640 Medium, (ATCC 30–2001) supplemented with fetal bovine serum (ATCC 30–2020) to a final concentration of 10%. Cells were maintained at 37 °C with 5% carbon dioxide (CO_2_) and were sub-cultured every 2 to 3 days, per ATCC recommended procedures using 0.25% (w/v) Trypsin-0.53 mM EDTA solution (ATCC 30–2101), until appropriate densities were reached. HCC1395BL; B lymphoblast; Epstein-Barr virus (EBV) transformed; Human (*Homo sapiens*) cells (expanded from ATCC CRL-2325) were cultured in ATCC-formulated Iscove’s Modified Dulbecco’s Medium, (ATCC Catalog No. 30–2005) supplemented with fetal bovine serum (ATCC 30–2020) to a final concentration of 20%. Cells were maintained at 37 °C with 5% CO_2_ and were sub-cultured every 2 to 3 days, per ATCC recommended procedures, using centrifugation with subsequent resuspension in fresh medium until appropriate densities were reached. Final cell suspensions were spun down and re-suspended in PBS for nucleic acid extraction.

All cellular genomic material was extracted using a modified Phenol- Chloroform-Iso-Amyl alcohol extraction approach. Essentially, cell pellets were re-suspended in TE, subjected to lysis in a 2% TritonX-100/0.1% SDS/0.1 M NaCl/10 mM Tris/1 mM EDTA solution and were extracted with a mixture of glass beads and Phenol- Chloroform-Iso-Amyl alcohol. Following multiple rounds of extraction, the aqueous layer was further treated with Chloroform-IAA and finally underwent RNases treatment and DNA precipitation using sodium acetate (3 M, pH 5.2) and ice-cold Ethanol. The final DNA preparation was re-suspended in TE and stored at −80 °C until use.

### FFPE processing and DNA extraction

Please see Online methods in our companion paper^2^ for details.

### Illumina WGS library preparation

The TruSeq DNA PCR-Free LT Kit (Illumina, FC-121-3001) was used to prepare samples for whole genome sequencing. WGS libraries were prepared at six sites with the TruSeq DNA PCR-Free LT Kit according to the manufacturers’ protocol. The input DNA amount for WGS library preparation with fresh DNA for TruSeq-PCR-free libraries was 1 ug unless otherwise specified. All sites used the same fragmentation conditions for WGS by using Covaris with targeted size of 350 bp. All replicated WGS were prepared on a different day.

The concentration of the TruSeq DNA PCR-Free libraries for WGS was measured by qPCR with the KAPA Library Quantification Complete Kit (Universal) (Roche, KK4824). The concentration of all the other libraries was measured by fluorometry either on the Qubit 1.0 fluorometer or on the GloMax Luminometer with the Quant-iT dsDNA HS Assay kit (ThermoFisher Scientific, Q32854). The quality of all libraries was assessed by capillary electrophoresis either on the 2100 Bioanalyzer or TapeStation instrument (Agilent) in combination with the High Sensitivity DNA Kit (Agilent, 5067-4626) or the DNA 1000 Kit (Agilent, 5067-1504) or on the 4200 TapeStation instrument (Agilent) with the D1000 assay (Agilent, 5067–5582 and 5067–5583).

For the WGS library preparation from cross-site study, the sequencing was performed at six sequencing sites using three different Illumina platforms including HiSeq 4000 instrument at 2 × 150 bases read length with HiSeq 3000/4000 SBS chemistry (cat# FC-410-1003), and on a NovaSeq instrument at 2 × 150 bases read length using the S2 configuration (cat#PN 20012860), or on a HiSeq X Ten at 2 × 150 bases read length using the X10 SBS chemistry (cat# FC-501-2501). Sequencing was performed following the manufacturer’s instructions.

For the comparison study of WGS library protocol using different input DNA amounts, Illumina TruSeq DNA PCR-free protocol used 250 ng input DNA, Illumina TruSeq Nano protocol libraries were prepared with 1 ng, 10 ng, and 100 ng input DNA amounts. Illumina Nextera Flex libraries were prepared with 1 ng, 10 ng, and 100 ng input DNA amounts. These libraries sequenced at two sequencing sites using two different Illumina platforms including HiSeq 4000 instrument (Illumina) at 2 × 150 bases read length with HiSeq 3000/4000 SBS chemistry (Illumina, FC-410-1003) and NovaSeq instrument (Illumina) at 2 × 150 bases read length using the S2 configuration (Illumina, PN 20012860). Sequencing was performed following the manufacturer’s instructions.

For the tumor purity study, 1 µg tumor:normal dilutions were made in the following ratios using Resuspension Buffer (Illumina): 1:0, 3:1, 1:1, 1:4, 1:9, 1:19 and 0:1. Each ratio was diluted in triplicate. DNA was sheared using the Covaris S220 to target a 350 bp fragment size (Peak power 140w, Duty Factor 10%, 200 Cycles/Bursts, 55 s, Temp 4 °C). NGS library preparation was performed using the Truseq DNA PCR-free protocol (Illumina) following the manufacturer’s recommendations. The sample purity WGS libraries were sequenced on a HiSeq 4000 instrument (Illumina) at 2 × 150 bases read length with HiSeq 3000/4000 SBS chemistry (Illumina, FC-410-1003). Sequencing was performed following the manufacturer’s instructions.

### Whole exome library construction and sequencing

SureSelect Target Enrichment Reagent kit, PTN (Part No G9605A), SureSelect Human All Exon v6 + UTRs (Part No 5190–8881), Herculase II Fusion DNA Polymerase (Part No 600677) from Agilent Technologies and Ion Xpress Plus Fragment kit (Part No 4471269, Thermo Fischer Scientific Inc) were combined to prepare library according to the manufacturer’s guidelines (User guide: SureSelect Target Enrichment System for Sequencing on Ion Proton, Version C0, December 2016, Agilent Technologies). Prior, during and after library preparation the quality and quantity of genomic DNA (gDNA) and/or libraries were evaluated applying QubitTM fluorometer 2.0 with dsDNA HS Assay Kit (Thermo Fischer Scientific Inc) and Agilent Bioanalyzer 2100 with High Sensitivity DNA Kit (Agilent Technologies).

WES libraries were sequenced at six sequencing sites with two different Illumina platforms, Hiseq 4000 instrument (Illumina) at 2 × 150 bases read length with HiSeq 3000/4000 SBS chemistry (Illumina, FC-410-1003) and Hiseq 2500 (Illumina) at 2 × 100 bases read length with HiSeq 2500 chemistry (Illumina, FC-401-4003). Sequencing was performed following the manufacturer’s instructions.

### Whole genome FFPE sample library preparation and sequencing

For the FFPE WGS study, NEBNext Ultra II (NEB) libraries were prepared according to the manufacturer’s instructions. However, input adjustments were made according to the dCq obtained for each sample using the TruSeq FFPE DNA Library Prep QC Kit (Illumina) to account for differences in sample amplifiability. A total of 33 ng of amplifiable DNA was used as input for each sample.

FFPE WGS libraries were sequenced on two different sequencing canters on Hiseq 4000 instrument (Illumina) at 2 × 150 bases read length with HiSeq 3000/4000 SBS chemistry (Illumina, FC-410-1003). Sequencing was performed following the manufacturer’s instructions.

### Whole exome FFPE sample library preparation and sequencing

For the FFPE study, SureSelect (Agilent) WES libraries were prepared according to the manufacturer’s instructions for 200 ng of DNA input, including reducing the shearing time to four minutes. Additionally, the adaptor-ligated libraries were split in half prior to amplification. One half was amplified for 10 cycles and the other half for 11 cycles to ensure adequate yields for probe hybridization. Both halves were combined after PCR for the subsequent purification step.

FFPE WES libraries were sequenced on at two sequencing sites with different Illumina platforms, Hiseq 4000 instrument (Illumina) at 2 × 150 bases read length with HiSeq 3000/4000 SBS chemistry (Illumina, FC-410-1003) and Hiseq 2500 (Illumina) at 2 × 100 bases read length with HiSeq 2500 chemistry (Illumina, FC-401-4003). Sequencing was performed following the manufacturer’s instructions.

### PacBio library preparation and sequencing

15 ug of material was sheared to 40 kbp with Megarupter (Diagenode). Per the Megarupter protocol the samples were diluted to <50 ng/ul. A 1x AMPure XP bead cleanup was performed. Samples were prepared as outlined on the PacBio protocol titled “Preparing >30 kbp SMRTbell Libraries Using Megarupter Shearing and Blue Pippin Size-Selection for PacBio RS II and Sequel Systems.” After library preparation, the library was run overnight for size selection using the Blue Pippin (Sage). The Blue Pippin was set to select a size range of 15–50 kbp. After collection of the desired fraction, a 1x AMPure XP bead cleanup was performed. The samples were loaded on the PacBio Sequel (Pacific Biosciences) following the protocol titled “Protocol for loading the Sequel.” The recipe for loading the instrument was generated by the Pacbio SMRTlink software v5.0.0. Libraries were prepared using Sequel chemistry kits v2.1, SMRTbell template kit 1.0 SPv3, magbead v2 kit for magbead loading, sequencing primer v3, and SMRTbell clean-up columns v2. Libraries were loaded at between 4 pM and 8 pM.Sequencing was performed following the manufacturer’s instructions.

### 10X Genomics Chromium genome library preparation and sequencing

Sequencing libraries were prepared from 1.25 ng DNA using the Chromium Genome Library preparation v2 kit (10X Genomics, cat #120257/58/61/62) according to the manufacturer’s protocol (#CG00043 Chromium Genome Reagent Kit v2 User Guide). The quality of the libraries was evaluated using the TapeStation D1000 Screen Tape (Agilent). The adapter-ligated fragments were quantified by qPCR using the library quantification kit for Illumina (KK4824, KAPA Biosystems) on a CFX384Touch instrument (BioRad) prior to cluster generation and sequencing. Chromium libraries were sequenced on a HiSeq X Ten or a HiSeq 4000 instrument at 2 × 150 base pair (bp) read length and using sequencing chemistry v2.5 or HiSeq 3000/4000 SBS chemistry (Illumina, cat# FC-410-1003) across five sequencing sites.

Sequencing was performed following the manufacturer’s instructions.

### AmpliSeq library construction and sequencing

AmpliSeq libraries were prepared in triplicate and prepared as specified in the Illumina protocol (Document # 1000000036408 v04) following the two oligo pools workflow with 10 ng of input genomic DNA per pool. The number of amplicons per pool was 1517 and 1506 respectively. The libraries were quality-checked using an Agilent Tapestation 4200 with the DNA HS 1000 kit and quantitated using a Qubit 3.0 and DNA high sensitivity assay kit. The libraries were applied to a MiSeq v2.0 flowcell. They were then amplified and sequenced with a MiSeq 300 cycle reagent cartridge with a read length of 2 × 150 base pair (bp). The MiSeq run produced 7.3 Gbp (94.5%) at ≥Q30. The total number of reads passing filter was 47,126,128 reads.

### Whole exome library Ion platform sequencing

SureSelect Target Enrichment Reagent kit, PTN (Part No G9605A), SureSelect Human All Exon v6 + UTRs (Part No 5190–8881), Herculase II Fusion DNA Polymerase (Part No 600677) from Agilent Technologies and Ion Xpress Plus Fragment kit (Part No 4471269, Thermo Fisher Scientific Inc) were combined to prepare libraries according to the manufacturer’s guidelines (User guide: SureSelect Target Enrichment System for Sequencing on Ion Proton, Version C0, December 2016, Agilent Technologies). Prior, during, and after library preparation the quality and quantity of genomic DNA (gDNA) and/or libraries were evaluated applying QubitTM fluorometer 2.0 with dsDNA HS Assay Kit (Thermo Fisher Scientific Inc) and Agilent Bioanalyzer 2100 with High Sensitivity DNA Kit (Agilent Technologies).

For sequencing the WES libraries, the Ion S5 XL Sequencing platform with Ion 540-Chef kit (Part No A30011, Thermo Fisher Scientific Inc) and the Ion 540 Chip kit (Part No A27766, Thermo Fisher Scientific Inc) were used. One sample per 540 chip was sequenced, generating up to 60 million reads with average length of 200 bp.

### 10X Genomics Single Cell CNV library construction, sequencing and analysis

HCC1395 and HCC1395 BL were cultured as described above. 500,000 cells of each culture were suspended in 1 mL suspension medium (10% DMSO in cell culture medium). Cells were harvested the next day for single-cell copy number variation (CNV) analysis via the 10X Genomics Chromium Single Cell CNV Solution (Protocol document CG000153) produces Single Cell DNA libraries ready for Illumina sequencing according to manufacturer’s recommendations. Libraries were sequenced on a HiSeq 4000 instrument at 2 × 150 base pair (bp) read length and using sequencing chemistry v2.5 or HiSeq 3000/4000 SBS chemistry (Illumina, cat# FC-410-1003). Demultiplex BCL from sequencing run and Copy Number Variation analysis were performed using 10X Genomics Cell Ranger DNA version 1.1 software. CNV and heterogeneity visualization analysis was performed via 10X Genomics Loupe scDNA browser.

### Affymetrix Cytoscan HD microarray

DNA concentration was measured spectrophotometrically using a Nanodrop (Life technology), and integrity was evaluated with a TapeStation 4200 (Agilent). Two hundred and fifty nanograms of gDNA were used to proceed with the Affymetrix CytoScan Assay kit (Affymetrix). The workflow consisted of restriction enzyme digestion with Nsp I, ligation, PCR, purification, fragmentation, and end labeling. DNA was then hybridized for 16 hr at 50 °C on a CytoScan array (Affymetrix), washed and stained in the Affymetrix Fluidics Station 450 (Affymetrix), and then scanned with the Affymetrix GeneChip Scanner 3000 G7 (Affymetrix). Data were processed with ChAS software (version 3.3). Array-specific annotation (NetAffx annotation release 36, built with human hg38 annotation) was used in the analysis workflow module of ChAS. Karyoview plot and segments data were generated with default parameters.

### Reference genome

The reference genome we used was the decoy version of the GRCh38/hg38 human reference genome (https://gdc.cancer.gov/about-data/data-harmonization-and-generation/gdc-reference-files; GRCh38.d1.dv1.fa), which was utilized by the Genomic Data Commons (GDC).

The gene annotation GTF file was downloaded from the 10X website as refdata-cellranger-GRCh38-1.2.0.tar.gz, which corresponds to the GRCh38 genome and Ensmebl v84 transcriptome.

All the following bioinformatics data analyses are based on the above reference genome and gene annotation.

### Preprocessing and alignment of WGS Illumina data

For each of the paired-end read files (i.e., FASTQ 1 and 2 files) generated by Illumina sequencers (HiSeq, NovaSeq, X Ten platforms), we first trimmed low-quality bases and adapter sequences using Trimmomatic^4^. The trimmed reads were mapped to the human reference genome GRCh38 (see the read alignment section) using BWA MEM (v0.7.17)^5^ in paired-end mode and bwa-mem was run with the –M flag for downstream Picard^6^ compatibility.

Post alignment QC was performed based both FASTQ on BWA alignment BAM files, the read quality and adapter content were reported by FASTQC^7^ software. The genome mapped percentages and mapped reads duplication rates calculated by BamTools (v2.2.3) and Picard (v1.84). The genome coverage and exome target region coverages as well as mapped reads insert sizes, and G/C contents were profiled using Qualimap(v2.2)^8^ and custom scripts. Preprocessing QC reports were generated during each step of the process. MultiQC(v1.9)^9^ was run to generate an aggregated report in html format. A standard QC metrics report was generated from a custom script. The preprocessing and alignment QC analysis pipeline is described in Suppl. Figure 1a.

### Preprocessing and alignment of WES Illumina data

For each of the paired-end read files generated by Illumina sequencers (HiSeq 2500, HiSeq 4000 platforms), we first trimmed low-quality bases and adapter sequences using Trimmomatic. The trimmed reads were mapped to the human reference genome GRCh38 (see the read alignment section) using BWA MEM (v0.7.17) in paired-end mode. We calculated on-target rate based on the percentage of mapped reads that were overlap the target capture bait region file (target.bed). The post alignment QC methods are same as WGS Illumina data pre-processing.

### DNA damage estimate for WGS, WES and FFPE samples

The DNA Damage Estimator(v3)^10^ was used to calculate the GIV score based on an imbalance between R1 and R2 variant frequency of the sequencing reads to estimate the level of DNA damage that was introduced in the sample/library preparation processes. GIV score above 1.5 is defined as damaged. At this GIV score, there are 1.5 times more variants on R1 than on R2. Undamaged DNA samples have a GIV score of 1.

### Preprocessing and alignment of PacBio data

PacBio raw data were merged bam files using SMRTlink tool v6.0.1. which used minimap2^11^ as default aligner. The non-human reads were removed and minimap BAM files were used for downstream analysis. Duplicate reads were mark and removed from PBSV alignment bases on the reads coming from the same ZMW, the base pair tolerance was set to 100 bp to remove the duplicated reads. The preprocessing and alignment QC analysis pipeline for PacBio data is described in Suppl. Figure 1b.

### Genome coverage profiling

We used indexcov^12^ to estimate coverage from the Illumina whole genome sequencing library cross-site comparison data set. The bam file for each library used as input to indexcov to generate a linear index for each chromosome indicating the file (and virtual) offset for every 16,384 bases in that chromosome. This gives the scaled value for each 16,384-base chunk (16KB resolution) and provides a high-quality coverage estimate per genome. The output is scaled to around 1. A long stretch with values of 1.5 would be a heterozygous duplication; a long stretch with values of 0.5 would be a heterozygous deletion.

### Preprocessing and alignment of 10X Genomics WGS data

The 10X Genomics Chromium fastq files were mapped and reads were phased using LongRanger to the hg38/GRCh38 reference genome using the LongRanger v2.2.2 pipeline [https://genome.cshlp.org/content/29/4/635.full]. The linked-reads were aligned using the Lariat aligner^13^, which uses BWA MEM to generate alignment candidates, and duplicate reads are marked after alignment. Linked-Read data quality was assessed using the 10X Genome browser Loupe. MultiQC(v1.9) was run to generate an aggregated report in html format. A standard QC metrics report was generated from a custom script. The preprocessing and alignment QC analysis pipeline is described in Suppl. Figure 1a.

### Preprocessing and alignment of Ion Torrent data

Raw reads were first filtered for low-quality reads and trimmed to remove adapter sequences and low-quality bases. This step was performed using the BaseCaller module of the Torrent SuitTM software package v5.8.0 (Thermo Fischer Scientific Inc). Low-quality reads were retained from further analysis in the raw signal processing stage. Low-quality bases were trimmed from the 5′ end if the average quality score of the 16-base window fell below 16 (Phred scale), cleaving 8 bases at once. Processed reads were mapped to the GRCh38 reference genome by TMAP module of the Torrent Suite software package using the default map4 algorithm with recommended settings. Picard (v1.84) was then used to mark PCR and optical duplicates on the BAM files.

### Preprocessing and alignment for AmpliSeq 

Low-quality bases and adapter sequences were trimmed with Trimmomatic. The trimmed reads were mapped to the human reference genome GRCh38 (see the read alignment section) using BWA MEM (v0.7.17) in paired-end mode. We calculated on-target rate based on the percentage of mapped reads that were overlap the target capture bait region file (target.bed). We counted the number of variant-supporting reads and total reads for each variant position with MQ ≥ 40 and BQ ≥ 30 cutoffs. The preprocessing and alignment QC analysis pipeline is described in Suppl. Figure 1a.

### Somatic variant analysis

Four somatic variant callers, MuTect2 (GATK 3.8-0)^14^, SomaticSniper (1.0.5.0)^15^, Strelka2 (2.8.4)^16^, and Lancet (1.0.7)^17^, which are readily available on the NIH Biowulf cluster, were run using the default parameters or parameters recommended by the user’s manual. Specifically, for MuTect2, we included flags for “-nct 1 -rf DuplicateRead -rf FailsVendorQualityCheck -rf NotPrimaryAlignment -rf BadMate -rf MappingQualityUnavailable -rf UnmappedRead -rf BadCigar”, to avoid the running exception for “Somehow the requested coordinate is not covered by the read”. For MuTect2, we used COSMIC v82 as required inputs. For SomaticSniper, we added a flag for “-Q 40 -G -L –F”, as suggested by its original author, to ensure quality scores and reduce likely false positives. For TNscope (201711.03), we used the version implemented in Seven Bridges’s CGC with the following command, “sentieon driver -i $tumor_bam -i $normal_bam -r $ref–algo TNscope–tumor_sample $tumor_sample_name–normal_sample $normal_sample_name -d $dbsnp $output_vcf”. For Lancet, we ran with 24 threads on the following parameters “–num-threads 24–cov-thr 10–cov-ratio 0.005–max-indel-len 50 -e 0.005”. Strelka2 was run with 24 threads with the default configuration. The rest of the software analyzed was run as a single thread on each computer node. All mutation calling on WES data was performed with the specified genome region in a BED file for exome-capture target sequences.

The high confidence outputs or SNVs flagged as “PASS” in the resulting VCF files were applied to our comparison analysis. Results from each caller used for comparison were all mutation candidates that users would otherwise consider as “real” mutations detected by this caller.

### GATK indel realignment and quality score recalibration

The GATK (3.8-0)-IndelRealigner was used to perform indel adjustment with reference indels defined in the 1000Genome project (ftp://ftp.1000genomes.ebi.ac.uk/vol1/ftp/technical/reference/GRCh38_reference_genome/other_mapping_resources/ALL.wgs.1000G_phase3.GRCh38.ncbi_remapper.20150424.shapeit2_indels.vcf.gz). The resulting BAM files were then recalibrated for quality with “BaseRecalibrator” and dbSNP build 146 as the SNP reference. Finally,”PrintReads” was used to generate recalibrated BAM files.

### Tumor ploidy and clonality analysis from whole genome and exome data

To estimate the HCC1395 cell line ploidy, we used PURPLE^18^ to determine the purity and copy number profile. To determine the clonality of HCC1395 and HCC1395 BL, we performed somatic SNV and CAN analysis using superFreq^19^. on capture WES datasets. Mapped and markDuplicate bam files of a pair of HCC1395 and HCC1395BL were used as input and bam files of the remaining replicates of the HCC1395BL library were used to filter background. Analysis was run using the superFreq default parameters. The clonality of each somatic SNV was calculated based on the VAF, accounting for local copy number. The SNVs and CNAs undergo hierarchical clustering based on the clonality and uncertainty across replicates for the tumor sample.

### Assessment of reproducibility and O_Score calculation

we established following formula to measure reproducibility based on the overlapping SNVs:

$${O}_{score}=\frac{{\sum }_{i=1}^{i\to n}\left(\left(\frac{i}{n}\right)\times {O}_{i}\right)}{{\sum }_{i=1}^{i\to n}{O}_{i}}$$

where n is the total number of VCF results in the pool set, i is the number of overlaps, *O*_*i*_ is the number of accumulated SNVs in the set with i number of overlapping.

## Data Records

All raw data (FASTQ files) are available on NCBI’s SRA database (SRP162370)^20^. The call set for somatic mutations in HCC1395, VCF files derived from individual WES and WGS runs, and source codes are available on NCBI’s ftp site (ftp://ftp-trace.ncbi.nlm.nih.gov/ReferenceSamples/seqc/Somatic_Mutation_WG/)^21^.

## Technical Validation

### Assessment of whole genome and exome sequencing data quality

Data set described in this paper was mainly used in our two companion studies, to assess the effect of variables during the process of WGS and WES, including biosample types, tumor content, library protocol and DNA inputs, sequencing site and replicates, reads coverage and bioinformatics tools, on the performance of cancer mutation detection^2^ and to characterize a pair of tumor-normal cell lines as community reference samples^3^.

The quality metrics including Total Reads, Total Reads After Trimming, Percent Total Reads after Trimming, Total Mapped Reads, Percent Total Mapped Reads (Trimmed), Percent Non-duplicated Reads (Mapped Trimmed), Mean Coverage Depth, Mean Coverage Depth SD, Percent of Coverages (> = 5x, > = 15x, > = 30x, > = 100x), Effect Mean Coverage Inside Target Region (Exome only), Percent of GC, Median Insert Size (Online-Only Tables 1–10).

For whole genome sequencing, fresh DNA samples were prepared using standard TruSeq PCR-free libraries prepared from 1000 ng input DNA. A total of 24 data sets were generated from six sequencing centers. There were three different Illumina sequencing platforms in the cross-platform comparison including HiSeq 4000, HiSeq X Ten, and NovaSeq 6000.

**Fig. 2:**
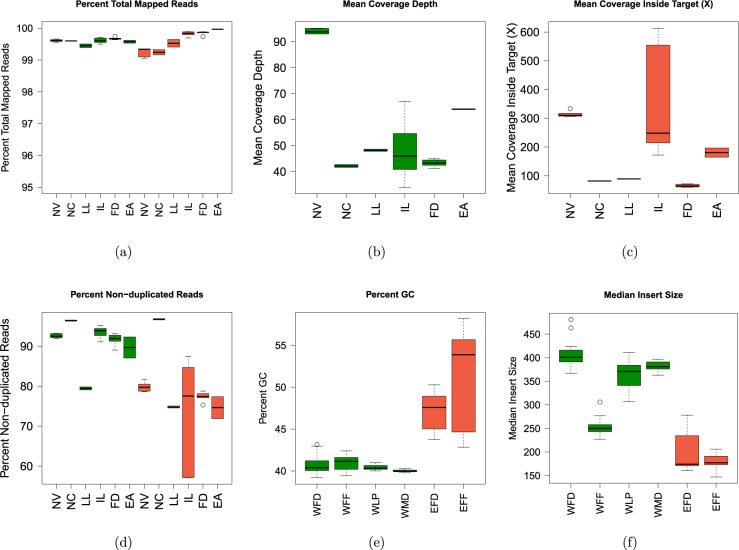
Overall data quality for WGS and WES data sets from Illumina platform. **(a)** Percentage of total reads mapped to reference genome (hg38) for WGS (Green) and WES (Red) across 6 sequencing sites. **(b)** Mean coverage depth for WGS libraries across 6 sequencing sites. (**c)** Mean coverage depth in target capture regions for WES libraries across 6 sequencing sites. **(d)** Percentage of non-duplicated reads mapped to reference genome across 6 sequencing sites. WGS (Green) and WES (Red). (**e)** Percent GC content from different library prep protocols. WGS (Green) and WES (Red). (**f)** Mean insert size distribution from different library prep protocols. WGS (Green) and WES (Red).

All sequencing centers and platforms produced high quality data as base call Phred quality scores above Q30, and greater than 99% of reads mapped to the reference genome (Fig. 2a). The variation was observed in read coverage which was driven by sequencing platform yield differences as well as sequencing library pooling variations. Most sequencing sites produced genome coverage 50X (1,250 millions pair-end reads) per library, one sequencing site targeted about 100X (2,500 millions pair-end reads) per genome sequencing depth (Fig. 2b, Suppl. Figure 2a). For whole exome sequencing, SureSelect Target Enrichment Reagent kit, PTN (Part No G9605A), SureSelect Human All Exon v6 and SureSelect Human All Exon v6 + UTRs were used by six sequencing centers. Illumina Hiseq 4000, Illumina Hiseq 3000/4000, and Illumina Hiseq 2500 were used. Sequencing quality from all sequences are high with greater than 99.1% of reads mapped to reference genome across sites. The variation was also observed in read coverage, most sequencing sites produced exome region on-target coverage 100X per library, and two sequencing sites targeted about 300X and 550X per genome sequencing depth (Fig. 2c). When comparing WGS to WES libraries for the percentages of non-duplicated reads, all WGS libraries have consistently high percentages of non-duplicate reads, which indicates higher library complexity of WGS libraries than the targeted captures. In addition, there are much high variations in targeted exome capture libraries(Fig. 2d).

To determine if the quality of sequencing data was substantially different between different protocols, we also compared fresh DNA vs. FFPE DNA, different library protocols and input DNA amount, as well as mixture tumor DNA and normal DNA for profiling the tumor purity effect. Among the WGS libraries prepared using fresh cells, insert size distribution and G/C content were uniform (40–43% G/C). WES libraries have higher GC content (47.2% for fresh cells libraries, 51.1% for FFPE libraries) as well as higher variation (Fig. 2e). All of the WGS libraries had very low adapter contamination (<0.5%) (Suppl. Figure 2b), while WES libraries have higher adapter content due to smaller DNA fragment insert sizes (Fig. 2f). WES library sizes are between 150 bps –280 bps for fresh cells. FFPE WGS libraries all have much shorter libraries sizes (225–300 bps) than fresh DNA prepared WGS libraries (360–480 bps). The libraries with higher adapter contamination also had much higher G/C content compared with the rest of the WES libraries (Fig. 2e). When comparing library preparation kits across different DNA inputs across TruSeq PCR-free (1000 ng), TruSeq-Nano, and Nextera Flex libraries prepared with 250, 100, 10, or 1 ng of DNA input, the percentage of non-redundant reads was very low (<20%) for TruSeq-Nano with 1 ng input, due to PCR amplification of a low input amount of DNA; higher input amount libraries have better performance; for the same input amount, Nextera Flex libraries have less variation and higher percentages of non-duplicated reads (Suppl. Figure 2c). We conclude the Nextera Flex library protocol might be a better option for low input DNA library preparation. The average GC% for WES and WGS samples are 48% and 41% respectively (Fig. 2e). However, from the binned GC and sequence coverage plots (Suppl. Figure 3a,b), we observed a higher sequencing coverage bias in very low GC (<25%) and very high GC content (>70%) in WES data. WGS showed more uniformed coverage across the spectrum of GC content except the extremely high or low GC content. This was due to different target capture affinity between probes and target DNA fragments. Extremely low or high GC content would impact binding affinity and thus can be captured less efficiently. This has been reported in the previous study^22^. As a result, WES reads would have overall higher coverage bias in very low GC and very high GC content regions.

### Assessment of reference sample sequencing coverage and genome heterogeneity

**Fig. 3:**
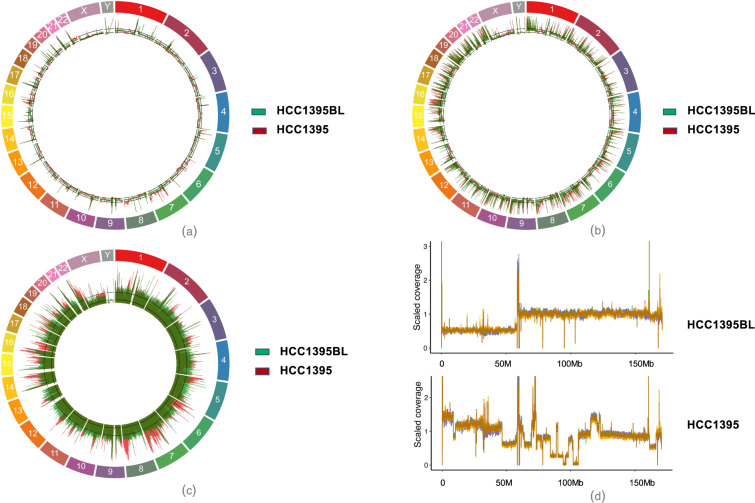
Genome coverage from WGS data from three technologies including Illumina, PacBio, and 10X Genomics. Outer rainbow color track: chromosomes, red track: HCC1395, green track: HCC1395BL. **(a)** Genome coverage from WGS data by reads from Illumina platform. **(b)** Genome coverage from WGS data by reads from 10X Chromium linked-read technology **(c)** Genome coverage from WGS data by reads from PacBio platform. **(d)** Genome coverage plots generated using Indexcov software for whole genome sequencing cross-site comparison libraries. The estimated coverages along chromosome 6 for HCC1395BL (top) and HCC1395 (bottom) are shown. The net loss of one copy of the short-arm of chr6 is shown for HCC1395BL on top. For tumor HCC1395 cell line, there are many copy number gain or loss as shown in bottom of the read coverage plot for chromosome 6.

We chose 26 replicates of HCC1395 and HCC1395BL data sets, which were libraries prepared using the Ilumina TruSeq DNA PCR free (1000 ng) protocol and sequenced on Illumina HiSeq and NovaSeq. Each library was ranged from 50X to 100X genome coverage (Fig. 3a, Suppl. Figure 4a). The percentage of genome coverage with less than 5X is 0.9–7.7% (Online-Only Table 1). We also compared fresh DNA vs. FFPE DNA, the FFPE WGS libraries have 50X to 100X genome coverage (Suppl. Figure 4b) and the percentage of genome coverage with less than 5X is 6.3–7.6% (Online-only Table 2). For 10X Chromium libraries, each library has 45X–120X genome coverage (Figs. 3b), 6.4–7.3% of genome regions have read coverage less than 5X (Online-only Table 7). 10X Chromium linked read technology produced input DNA molecule length in the range between 54–77 kb. The site-to-site variation was due to sequencing depth differences. For WES samples, the target region has nearly 100% coverage by sequencing reads, however, we observed high variation in the sequencing coverage within each replicate as well as among replicates (Suppl. Figure 4c,d).

In addition, we generated two PacBio libraries with 40X of genome coverage from subreads. Long reads improve the map ability in repetitive genome regions where short-reads might fail to map correctly. PacBio long-read sequencing may cover the genomic regions where short reads cannot be mapped especially in the high GC/AT or low complexity genomic regions (Fig. 3c). However, its higher sequencing error rate than short-read sequencing affects the accuracy for the low-frequency somatic mutation discovery. The variation in genome coverage might be due to differences in sequencing technologies. From the study, short reads WGS has better uniform coverage compared to long reads. However, there is better coverage for certain genomic regions in long-read technologies; most noticeable are the highly repetitive regions, extreme GC regions, or around the centromere regions.

The Indexcov scaled read depth on reference genome for HCC1395 and HCC1395BL showed HCC1395 harboring many Copy Number Variation (gain or loss) events on every chromosome; HCC1395BL genome largely remains diploid except for chr6 and chr16 and chrX. Figure 3d showed read coverage on chromose 6, a net loss of one copy of the short-arm of chr6 for HCC1395BL and large copy number variations for HCC1395. Cytogenetic analysis with Affymetrix Cytoscan HD microarray confirms the Cytogenetic view of HCC1395 which harbors many copy numbers gains or losses; Cytogenetic view of HCC1395BL confirms the losses of chr6p, chr16q, and chrX^3^.

For HCC1395 cell line, the tumor purity and ploidy estimated from Illumina WGS data set (Suppl. Figure 5a) using PURPLE software showed the tumor purity is 99% and the ploidy is around 2.85. Cell ploidy histogram from 10X Chromium single cell CNV data set (Suppl. Figure 5b) displayed the vast majority of cells form a peak around ploidy 2.8. The analysis of 1270 cells for HCC1395 from 10X Single Cell CNV data set also revealed numerous chromosome gains and losses events (Suppl. Figure 5c) consistently in sub-populations of cells, which confirmed HCC1395 is a heterogeneous cell line.

### Assessment DNA damage artifacts

A previous study has revealed that DNA damage accounts for the majority of the false calls for the so-called low-frequency (1–5%) genetic variants in large public databases^10^. The DNA damage directly confounds the determination of somatic variants in those data sets. The Global Imbalance Value (GIV) score is commonly used to measure DNA damage based on an imbalance between paired-end sequencing R1 and R2 variant frequency^10^. GIV scores to capture the DNA damage due to the artifacts introduced during genomic library preparation, the combination of heat, shearing, and contaminates can result in the 8-oxoguanine base pairing with either cytosine or adenine, ultimately leading to G > T transversion mutations during PCR amplification^23^. In addition, Formaldehyde also causes the deamination of guanine. FFPE is known to cause G > T/C > A artifacts^24^.

**Fig. 4:**
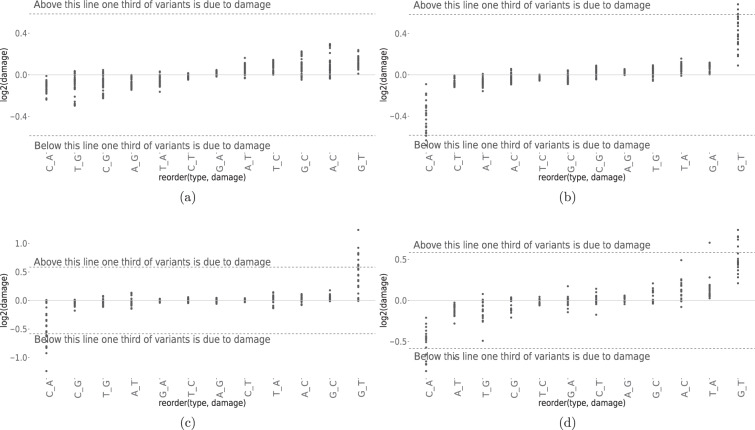
Evaluation of DNA damage for WGS and WES libraries. using GIV scores to capture the DNA damage due to the artifacts introduced during genomic library preparation. The estimation of damage is a global estimation based in an imbalance between R1 and R2 variant frequency. GIV score above 1.5 is defined as damaged. Undamaged DNA samples have a GIV score of 1. **(a)** DNA damage estimated for fresh cell prepared DNA for WGS Illumina libraries across different sites. **(b)** DNA damage estimated for FFPE WGS Illumina libraries. **(c)** DNA damage estimated for fresh cells prepared DNA for WES Illumina libraries across different sites **(d)** DNA damage estimated for FFPE WES Illumina libraries.

We calculated GIV score to monitor DNA damage in Illumina WGS and WES runs for both fresh DNA libraries as well as FFPE libraries. We found lower GIV scores for the G > T/C > A mutation pairs in fresh DNA WGS libraries (Fig. 4a) than FFPE WGS libraries (Fig. 4b). In addition, both fresh cell DNA WES (Fig. 4c) and FFPE WES Libraries (Fig. 4d) all showed increased GIV scores for the G > T/C > A mutation pairs relative to WGS libraries. The GIV for G > T/C > A scores was inversely correlated with insert fragment sizes, and it is positively correlated to DNA shearing time (Suppl. Figure 6a–c); WES libraries have consistently shorter library insert sizes than all WGS library sizes (Fig. 2f, Suppl. Figure 6a). Thus, the GIV of G > T/C > A is a good indicator of DNA damage introduced during genomic library preparation. We observe the libraries have high G > T/C > A GIV scores also have a higher percentage of C/A mutation called in WES from private mutation calls which are not shared among replicates as displayed in Suppl. Figure 6d. Therefore, in order to improve cancer genomic variant call accuracy, effective mitigation strategies to improve library preparation methods, or software tools to detect and remove the DNA damage mutation calls are essential.

### Assessment reproducibility of somatic mutation calling from WES and WGS data sets

**Fig. 5:**
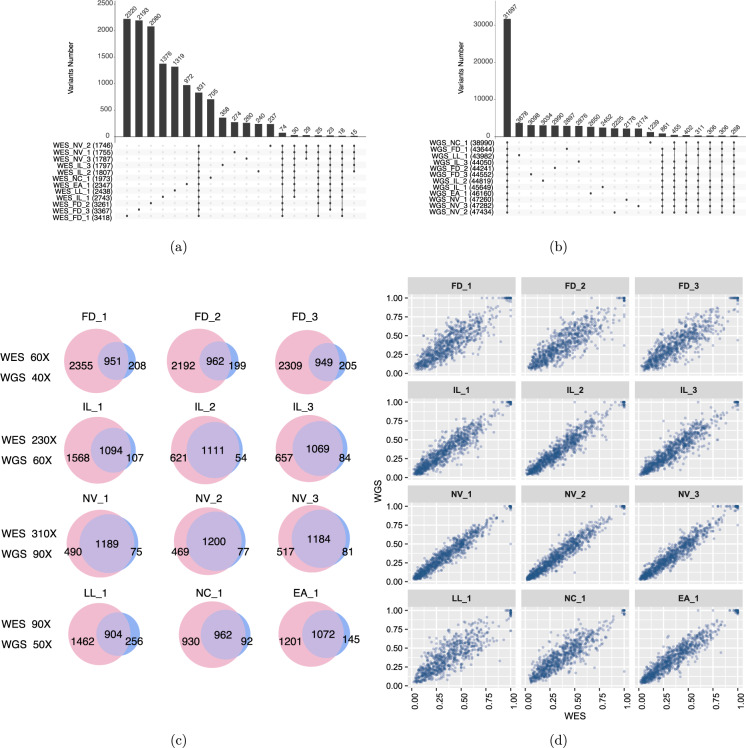
Reproducibility of somatic mutation calling from WES and WGS. The reproducibility UpSet plots for 12 repeated WES **(a)** and WGS runs **(b)**. The number in each plot represents the reproducibility across the different replicates. **(c)** SNVs/indels calling concordance between WES and WGS from twelve repeated runs. For direct comparison, SNVs/indels from WGS runs were limited to genomic regions defined by an exome capturing kit (SureSelect V6 + UTR). WES is shown on the left in the Venn diagram and WGS is on the right. Shown coverage depths for WES and WGS were effective mean sequence coverage on exome region, i.e. coverage by total number of mapped reads after trimming. **(d)** Correlation of MAF in overlapping WGS and WES SNVs/indels from repeated runs.

To assess the concordance and reproducibility of the somatic variant detection with both WES and WGS, we compared 12 replicates of WGS and WES for the matched tumor and normal cell lines carried out at six sequencing centers. Using three mutation callers (MuTect2, Strelka2, and SomaticSniper) on alignments from three aligners (Bowtie2^25^, BWA MEM, and NovoAlign), we generated a total of 108 variant call files separately. We were able to assess inter- and intra-centers reproducibility of the WES and WGS using the 12 repeat runs. The Venn diagram is widely used to display concordance of mutation calling results from a small number of repeated analyses; however, this type of diagram is not suitable for large data sets. To address this challenge, we applied the “UpSet” plot to visualize the consistency of mutation called across all conditions. As shown at the top of each plot (Fig. 5a,b), we observed relatively more library-specific variants in the WES plots. In contrast, majority of called mutations were shared across all 12 WGS (Fig. 5b). Therefore, calling results from WES tended to have more inconsistent SNV calls than those from WGS, indicating that WES results were less consistent than WGS results (Fig. 5a,b). Here we also introduced the O_Score, a metric to measure reproducibility of repeated analyses (see Methods). O_Scores for WES runs were not only significantly lower than WGS runs, but also more variable (Suppl. Figure 7). In addition, we measured reproducibility between replicates of WGS runs from both NovaSeq and HiSeq platforms to assess cross-platform variation. Both platforms were remarkably similar in terms of reproducibility, indicating that results from HiSeq and NovaSeq are comparable^2^. Overall, we observed the cross-center and cross-platform variations for WGS were very small, indicating that all individual NGS runs, regardless of sequencing centers or NGS platforms, detected most “true” mutations consistently for WGS runs.

We also computed SNVs/indels calling concordance between WES and WGS from twelve replicates. For direct comparison, SNVs/indels from WGS runs were limited to genomic regions defined by an exome capturing protocol (SureSelect V6 + UTR). WGS has a smaller number of private calls for each sample than WES (Fig. 5c). We observed the overlap between the WES and WGS improved as sequencing depth increased. Moreover, the correlation of MAF in overlapping WGS and WES SNVs/indels from replicates are positively correlated with higher sequencing depth (Fig. 5d). This indicates the benefit of high read coverage not only improves the detection sensitivity of mutations with low MAF, but also increases reproducibility of the calling sets. Overall, our results indicate the inter-center variations for WES were larger than inter-center variations for WGS, whereas the difference between intra-center variation between WES and WGS was not significant. As a result, WGS had much less inter-center variation and thus provided better reproducibility than WES for cancer genomic variants detection.

## Supplementary information

Supplementary Figures

## Data Availability

All codes used in processing the whole genome, exome-seq and RNA-seq data are available to download at NCBI’s ftp site (https://ftp-trace.ncbi.nlm.nih.gov/ReferenceSamples/seqc/Somatic_Mutation_WG/tools/NGS_Preprocessing_Pipeline)^21^.

## Online-Only Table

**Online-Only Table 1:** Whole Genome Sequencing (WGS) data sets for fresh DNA extracted from HCC1395BL and HCC1395 cell lines.

Sample ID	Sample	Biosample	Platform	Machine model	library Protocol (Input amount)	Total Reads	Total Reads After Trimming	Percent Total Reads after Trimming	Total Mapped Reads	Percent Total Mapped Reads (Trimmed)	Percent Non-duplicated Reads (Mapped Trimmed)	Mean Coverage Depth	Mean Coverage Depth SD	Percent of Coverage > = 5X	Percent of Coverage > = 15X	Percent of Coverage > = 30X	Percent GC	Median Insert Size
WGS_IL_N_1	HCC1395BL	Fresh cells	HiSeq	HiSeq 3000/4000	TruSeq DNA PCR Free (1000 ng)	1,922,974,720	1,921,659,788	99.93	1,913,037,464	99.55	95.20	58.98	351.92	93.68	93.12	87.97	39.70	419
WGS_IL_N_2	HCC1395BL	Fresh cells	HiSeq	HiSeq 3000/4000	TruSeq DNA PCR Free (1000 ng)	1,552,485,544	1,551,295,230	99.92	1,546,324,619	99.68	93.78	50.04	370.95	93.64	92.41	82.08	39.22	393
WGS_IL_N_3	HCC1395BL	Fresh cells	HiSeq	HiSeq 3000/4000	TruSeq DNA PCR Free (1000 ng)	1,672,746,794	1,672,206,086	99.97	1,664,765,047	99.56	94.50	50.79	384.54	93.66	92.53	82.59	39.18	402
WGS_IL_T_1	HCC1395	Fresh cells	HiSeq	HiSeq 3000/4000	TruSeq DNA PCR Free (1000 ng)	2,152,864,926	2,151,176,006	99.92	2,141,479,936	99.55	95.11	67.00	292.90	93.05	92.38	86.88	39.90	417
WGS_IL_T_2	HCC1395	Fresh cells	HiSeq	HiSeq 3000/4000	TruSeq DNA PCR Free (1000 ng)	1,742,944,436	1,741,213,400	99.90	1,735,985,947	99.70	92.73	56.10	288.78	92.99	91.40	80.83	39.63	395
WGS_IL_T_3	HCC1395	Fresh cells	HiSeq	HiSeq 3000/4000	TruSeq DNA PCR Free (1000 ng)	1,745,338,388	1,744,749,370	99.97	1,737,271,357	99.57	93.05	53.38	289.37	93.00	91.22	78.86	39.40	401
WGS_EA_N_1	HCC1395BL	Fresh cells	HiSeq	HiSeq X10	TruSeq DNA PCR Free (1000 ng)	1,740,311,982	1,737,882,004	99.86	1,731,224,725	99.62	87.04	64.22	337.23	93.65	93.31	90.53	40.05	412
WGS_EA_T_1	HCC1395	Fresh cells	HiSeq	HiSeq X10	TruSeq DNA PCR Free (1000 ng)	1,885,118,894	1,883,492,596	99.91	1,874,626,618	99.53	92.32	63.85	246.40	93.03	92.48	87.75	40.20	422
WGS_NC_N_1	HCC1395BL	Fresh cells	HiSeq	HiSeq 3000/4000	TruSeq DNA PCR Free (1000 ng)	1,232,116,762	1,231,187,894	99.93	1,226,338,013	99.61	96.27	42.69	52.04	99.09	92.12	79.19	42.95	417
WGS_NC_T_1	HCC1395	Fresh cells	HiSeq	HiSeq 3000/4000	TruSeq DNA PCR Free (1000 ng)	1,202,406,138	1,201,304,904	99.91	1,196,430,615	99.59	96.53	41.61	44.76	98.84	89.65	67.71	43.15	408
WGS_LL_N_1	HCC1395BL	Fresh cells	HiSeq	HiSeq 4000	TruSeq DNA PCR Free (1000 ng)	1,267,721,898	1,267,074,486	99.95	1,260,762,149	99.50	79.06	48.62	272.02	93.57	87.36	50.48	39.95	377
WGS_LL_T_1	HCC1395	Fresh cells	HiSeq	HiSeq 4000	TruSeq DNA PCR Free (1000 ng)	1,248,345,468	1,247,771,438	99.95	1,240,175,659	99.39	79.82	47.80	196.07	92.89	80.01	35.94	40.20	372
WGS_NV_N_1	HCC1395BL	Fresh cells	HiSeq	HiSeq 4000	TruSeq DNA PCR Free (1000 ng)	2,737,041,074	2,736,265,592	99.97	2,725,324,100	99.60	93.22	93.15	555.75	93.72	93.51	92.88	40.03	400
WGS_NV_N_2	HCC1395BL	Fresh cells	HiSeq	HiSeq 4000	TruSeq DNA PCR Free (1000 ng)	2,755,558,524	2,753,791,014	99.94	2,743,237,055	99.62	93.17	95.22	557.97	93.72	93.51	92.91	39.98	390
WGS_NV_N_3	HCC1395BL	Fresh cells	HiSeq	HiSeq 4000	TruSeq DNA PCR Free (1000 ng)	2,765,895,594	2,764,785,302	99.96	2,752,475,146	99.56	92.14	94.43	555.64	93.72	93.51	92.90	39.96	395
WGS_NV_T_1	HCC1395	Fresh cells	HiSeq	HiSeq 4000	TruSeq DNA PCR Free (1000 ng)	2,708,598,970	2,706,761,164	99.93	2,695,472,614	99.58	92.77	92.97	436.57	93.08	92.81	91.27	40.18	404
WGS_NV_T_2	HCC1395	Fresh cells	HiSeq	HiSeq 4000	TruSeq DNA PCR Free (1000 ng)	2,752,035,508	2,751,442,992	99.98	2,741,523,523	99.64	92.23	95.13	496.20	93.08	92.82	91.42	40.18	394
WGS_NV_T_3	HCC1395	Fresh cells	HiSeq	HiSeq 4000	TruSeq DNA PCR Free (1000 ng)	2,752,204,218	2,751,260,128	99.97	2,741,889,519	99.66	91.95	92.97	425.71	93.08	92.81	91.24	40.2	387
WGS_FD_N_1	HCC1395BL	Fresh cells	HiSeq	HiSeq X10	TruSeq DNA PCR Free (1000 ng)	1,140,214,238	1,139,328,958	99.92	1,135,160,204	99.63	92.15	42.43	219.92	93.56	92.44	82.02	40.15	367
WGS_FD_N_2	HCC1395BL	Fresh cells	HiSeq	HiSeq X10	TruSeq DNA PCR Free (1000 ng)	1,197,153,800	1,196,576,644	99.95	1,192,633,894	99.67	93.15	44.46	237.23	93.57	92.63	83.31	40.06	371
WGS_FD_N_3	HCC1395BL	Fresh cells	HiSeq	HiSeq X10	TruSeq DNA PCR Free (1000 ng)	1,174,631,698	1,174,023,848	99.95	1,170,212,823	99.68	92.75	43.43	230.61	93.57	92.55	82.72	40.1	368
WGS_FD_T_1	HCC1395	Fresh cells	HiSeq	HiSeq X10	TruSeq DNA PCR Free (1000 ng)	1,100,936,840	1,100,540,416	99.96	1,096,693,277	99.65	91.24	41.16	155.16	92.88	90.27	69.33	40.34	377
WGS_FD_T_2	HCC1395	Fresh cells	HiSeq	HiSeq X10	TruSeq DNA PCR Free (1000 ng)	1,136,080,154	1,135,450,092	99.95	1,131,808,658	99.68	91.65	43.07	160.70	92.88	90.65	72.60	40.43	375
WGS_FD_T_3	HCC1395	Fresh cells	HiSeq	HiSeq X10	TruSeq DNA PCR Free (1000 ng)	1,166,219,086	1,165,667,134	99.95	1,162,681,088	99.74	89.02	44.95	171.40	92.89	90.90	75.11	40.33	371
WGS_NS_N_1	HCC1395BL	Fresh cells	NovaSeq	NovaSeq 6000	TruSeq DNA PCR Free (1000 ng)	1,406,727,868	1,404,557,816	99.85	1,400,188,308	99.69	92.63	45.95	262.43	93.61	92.86	84.87	41.26	423
WGS_NS_N_2	HCC1395BL	Fresh cells	NovaSeq	NovaSeq 6000	TruSeq DNA PCR Free (1000 ng)	1,505,296,280	1,504,606,110	99.95	1,497,164,368	99.51	93.97	41.48	215.63	93.63	92.53	82.24	40.86	400
WGS_NS_N_3	HCC1395BL	Fresh cells	NovaSeq	NovaSeq 6000	TruSeq DNA PCR Free (1000 ng)	1,357,261,200	1,357,106,672	99.99	1,350,908,040	99.54	94.72	40.61	216.19	93.61	92.37	81.33	41.20	463
WGS_NS_N_4	HCC1395BL	Fresh cells	NovaSeq	NovaSeq 6000	TruSeq DNA PCR Free (1000 ng)	1,367,955,412	1,367,339,034	99.96	1,362,514,522	99.65	94.13	41.75	228.73	93.61	92.54	82.51	41.11	419
WGS_NS_N_5	HCC1395BL	Fresh cells	NovaSeq	NovaSeq 6000	TruSeq DNA PCR Free (1000 ng)	1,501,530,522	1,501,116,932	99.97	1,495,542,591	99.63	93.60	45.91	252.24	93.63	92.91	84.98	41.15	416
WGS_NS_N_6	HCC1395BL	Fresh cells	NovaSeq	NovaSeq 6000	TruSeq DNA PCR Free (1000 ng)	1,564,194,248	1,563,842,202	99.98	1,559,297,663	99.71	91.12	51.52	285.71	93.63	93.13	87.07	41.23	413
WGS_NS_N_7	HCC1395BL	Fresh cells	NovaSeq	NovaSeq 6000	TruSeq DNA PCR Free (1000 ng)	1,283,402,840	1,282,990,744	99.97	1,278,660,640	99.66	92.62	41.37	265.40	93.59	92.46	82.12	41.21	410
WGS_NS_N_8	HCC1395BL	Fresh cells	NovaSeq	NovaSeq 6000	TruSeq DNA PCR Free (1000 ng)	1,462,304,546	1,461,608,958	99.95	1,454,550,980	99.52	94.39	40.66	209.18	93.62	92.41	81.52	40.93	399
WGS_NS_N_9	HCC1395BL	Fresh cells	NovaSeq	NovaSeq 6000	TruSeq DNA PCR Free (1000 ng)	1,470,980,018	1,470,257,498	99.95	1,463,536,841	99.54	94.01	40.94	209.21	93.63	92.45	81.76	40.90	401
WGS_NS_T_1	HCC1395	Fresh cells	NovaSeq	NovaSeq 6000	TruSeq DNA PCR Free (1000 ng)	1,695,377,648	1,693,184,204	99.87	1,688,102,934	99.70	91.64	55.97	235.94	93.00	92.10	85.08	41.46	422
WGS_NS_T_2	HCC1395	Fresh cells	NovaSeq	NovaSeq 6000	TruSeq DNA PCR Free (1000 ng)	1,261,325,698	1,260,860,796	99.96	1,254,572,158	99.50	94.60	34.93	136.34	92.90	88.65	56.06	41.20	401
WGS_NS_T_3	HCC1395	Fresh cells	NovaSeq	NovaSeq 6000	TruSeq DNA PCR Free (1000 ng)	1,417,458,666	1,417,283,958	99.99	1,411,500,925	99.59	94.34	42.23	186.34	92.97	90.56	70.59	41.30	481
WGS_NS_T_4	HCC1395	Fresh cells	NovaSeq	NovaSeq 6000	TruSeq DNA PCR Free (1000 ng)	1,842,018,210	1,839,790,050	99.88	1,832,403,023	99.60	93.14	56.30	224.01	93.03	92.17	85.38	41.40	424
WGS_NS_T_5	HCC1395	Fresh cells	NovaSeq	NovaSeq 6000	TruSeq DNA PCR Free (1000 ng)	1,252,160,470	1,251,737,302	99.97	1,247,854,937	99.69	93.80	38.70	164.96	92.92	89.82	64.15	41.39	416
WGS_NS_T_6	HCC1395	Fresh cells	NovaSeq	NovaSeq 6000	TruSeq DNA PCR Free (1000 ng)	1,708,185,366	1,707,737,216	99.97	1,702,648,733	99.70	91.74	56.14	253.64	93.01	92.13	85.25	41.40	416
WGS_NS_T_7	HCC1395	Fresh cells	NovaSeq	NovaSeq 6000	TruSeq DNA PCR Free (1000 ng)	1,565,451,318	1,565,121,762	99.98	1,560,619,578	99.71	92.10	51.27	245.10	92.99	91.78	82.23	41.36	407
WGS_NS_T_8	HCC1395	Fresh cells	NovaSeq	NovaSeq 6000	TruSeq DNA PCR Free (1000 ng)	1,228,386,838	1,227,945,398	99.96	1,223,091,592	99.61	94.87	33.97	133.30	92.89	88.27	53.79	41.22	391
WGS_NS_T_9	HCC1395	Fresh cells	NovaSeq	NovaSeq 6000	TruSeq DNA PCR Free (1000 ng)	1,368,505,404	1,367,590,086	99.93	1,361,753,887	99.57	94.29	38.33	148.99	92.94	89.80	63.81	41.14	395

Libraries were made from TruSeq DNA PCR Free (1000 ng) library protocol and sequenced by six sequencing centers on Illumina HiSeq 3000/4000, HiSeq X10 and NovaSeq 6000 for cross-site comparision.

**Online-Only Table 2:** Whole Genome Sequencing (WGS) data sets for Formalin-Fixed Paraffin-Embedded (FFPE) process with fixation time of 1, 2, 6, or 24 hours for DNA extracted from HCC1395BL and HCC1395 cell lines.

Sample ID	Sample	Biosample	Platform	Machine model	library Protocol (Input amount	Total Reads	Total Reads After Trimming	Percent Total Reads after Trimming	Total Mapped Reads	Percent Total Mapped Reads (Trimmed)	Percent Non-duplicated Reads (Mapped Trimmed)	Mean Coverage Depth	Mean Coverage Depth SD	Percent of Coverage > = 5X	Percent of Coverage > = 15X	Percent of Coverage > = 30X	Percent GC	Median Insert Size
FFG_IL_N_1h	HCC1395BL	FFPE	HiSeq	HiSeq 4000	NEBNext Ultra II (200 ng)	1,765,128,404	1,764,489,856	99.96	1,755,040,098	99.46	81.33	60.73	275.63	93.45	92.21	86.08	42.39	263
FFG_IL_N_24h	HCC1395BL	FFPE	HiSeq	HiSeq 4000	NEBNext Ultra II (200 ng)	1,639,966,382	1,639,566,366	99.98	1,630,268,029	99.43	83.76	58.14	273.91	93.45	92.28	85.66	40.35	258
FFG_IL_N_2h	HCC1395BL	FFPE	HiSeq	HiSeq 4000	NEBNext Ultra II (200 ng)	2,068,216,980	2,067,386,842	99.96	2,058,632,971	99.58	76.51	73.91	352.49	93.54	92.83	89.46	42.15	251
FFG_IL_N_6h	HCC1395BL	FFPE	HiSeq	HiSeq 4000	NEBNext Ultra II (200 ng)	1,524,211,818	1,523,752,982	99.97	1,516,101,932	99.50	81.66	53.68	279.78	93.44	91.89	83.37	41.94	264
FFG_IL_T_1h	HCC1395	FFPE	HiSeq	HiSeq 4000	NEBNext Ultra II (200 ng)	2,624,805,980	2,622,947,802	99.93	2,604,551,075	99.30	76.27	89.63	321.88	92.93	92.30	89.27	39.73	277
FFG_IL_T_24h	HCC1395	FFPE	HiSeq	HiSeq 4000	NEBNext Ultra II (200 ng)	1,721,612,052	1,721,231,716	99.98	1,710,443,429	99.37	84.68	59.41	477.62	92.99	91.52	81.57	39.43	269
FFG_IL_T_2h	HCC1395	FFPE	HiSeq	HiSeq 4000	NEBNext Ultra II (200 ng)	1,962,242,886	1,961,844,318	99.98	1,953,044,006	99.55	82.43	69.99	319.98	92.88	91.50	84.05	39.61	265
FFG_IL_T_6h	HCC1395	FFPE	HiSeq	HiSeq 4000	NEBNext Ultra II (200 ng)	2,943,133,326	2,941,257,406	99.94	2,918,794,880	99.24	75.07	100.42	608.82	93.06	92.71	90.90	40.19	306
FFG_GZ_N_1h-B	HCC1395BL	FFPE	HiSeq	HiSeq 4000	NEBNext Ultra II (200 ng)	2,149,948,070	2,149,439,522	99.98	2,136,427,075	99.40	86.88	67.84	319.70	93.54	92.37	85.63	40.12	247
FFG_GZ_N_1h-F	HCC1395BL	FFPE	HiSeq	HiSeq 4000	NEBNext Ultra II (200 ng)	2,403,027,922	2,401,640,844	99.94	2,392,089,625	99.60	85.68	77.14	194.74	93.38	92.37	89.44	41.62	238
FFG_GZ_N_24h-B	HCC1395BL	FFPE	HiSeq	HiSeq 4000	NEBNext Ultra II (200 ng)	2,460,398,476	2,459,792,090	99.98	2,448,071,395	99.52	85.20	78.05	649.98	93.68	93.33	91.38	41.19	244
FFG_GZ_N_24h-C	HCC1395BL	FFPE	HiSeq	HiSeq 4000	NEBNext Ultra II (200 ng)	1,761,455,916	1,756,523,044	99.72	1,746,804,745	99.45	84.75	54.76	190.92	93.28	91.67	84.17	41.39	232
FFG_GZ_N_24h-F	HCC1395BL	FFPE	HiSeq	HiSeq 4000	NEBNext Ultra II (200 ng)	2,113,753,208	2,112,823,428	99.96	2,104,899,010	99.63	87.70	68.93	250.14	93.44	92.41	87.92	40.72	227
FFG_GZ_N_2h-A	HCC1395BL	FFPE	HiSeq	HiSeq 4000	NEBNext Ultra II (200 ng)	3,121,263,208	3,109,608,214	99.63	3,095,791,797	99.56	75.15	97.51	620.28	93.71	93.34	91.95	41.61	242
FFG_GZ_N_2h-E	HCC1395BL	FFPE	HiSeq	HiSeq 4000	NEBNext Ultra II (200 ng)	1,844,864,910	1,842,574,250	99.88	1,835,180,122	99.60	87.31	58.71	163.92	93.29	91.83	85.43	40.76	253
FFG_GZ_N_6h-A	HCC1395BL	FFPE	HiSeq	HiSeq 4000	NEBNext Ultra II (200 ng)	2,348,616,522	2,347,656,712	99.96	2,336,897,178	99.54	85.56	74.84	552.08	93.66	93.27	90.84	41.44	249
FFG_GZ_N_6h-E	HCC1395BL	FFPE	HiSeq	HiSeq 4000	NEBNext Ultra II (200 ng)	2,324,253,696	2,322,277,434	99.92	2,312,897,831	99.60	85.59	74.02	270.78	93.48	92.60	89.34	41.11	248
FFG_GZ_T_1h-A	HCC1395	FFPE	HiSeq	HiSeq 4000	NEBNext Ultra II (200 ng)	2,173,073,798	2,172,389,654	99.97	2,161,992,309	99.52	85.94	66.90	653.36	93.01	91.95	84.29	41.64	256
FFG_GZ_T_1h-B	HCC1395	FFPE	HiSeq	HiSeq 4000	NEBNext Ultra II (200 ng)	1,631,699,638	1,618,249,558	99.18	1,610,013,338	99.49	82.37	51.10	105.70	92.36	89.26	75.89	40.67	253
FFG_GZ_T_1h-E	HCC1395	FFPE	HiSeq	HiSeq 4000	NEBNext Ultra II (200 ng)	2,441,354,752	2,440,848,016	99.98	2,431,265,220	99.61	86.43	78.56	156.28	92.75	91.44	86.19	40.18	252
FFG_GZ_T_24h-B	HCC1395	FFPE	HiSeq	HiSeq 4000	NEBNext Ultra II (200 ng)	2,517,517,314	2,516,534,444	99.96	2,506,328,630	99.59	84.68	80.08	489.37	93.04	92.53	88.90	41.49	249
FFG_GZ_T_24h-F	HCC1395	FFPE	HiSeq	HiSeq 4000	NEBNext Ultra II (200 ng)	3,096,658,362	3,095,956,264	99.98	3,082,562,699	99.57	84.29	98.75	295.06	92.97	92.31	89.34	39.91	257
FFG_GZ_T_2h-A	HCC1395	FFPE	HiSeq	HiSeq 4000	NEBNext Ultra II (200 ng)	2,281,259,460	2,279,880,072	99.94	2,265,837,473	99.38	82.69	71.81	577.42	93.02	92.19	86.23	41.71	243
FFG_GZ_T_2h-B	HCC1395	FFPE	HiSeq	HiSeq 4000	NEBNext Ultra II (200 ng)	1,844,049,210	1,833,063,922	99.40	1,823,246,662	99.46	80.78	59.04	146.98	92.69	90.75	81.60	41.32	242
FFG_GZ_T_6h-A	HCC1395	FFPE	HiSeq	HiSeq 4000	NEBNext Ultra II (200 ng)	2,133,526,796	2,132,509,862	99.95	2,123,023,988	99.56	82.75	66.89	556.68	93.01	92.08	85.27	41.58	246
FFG_GZ_T_6h-B	HCC1395	FFPE	HiSeq	HiSeq 4000	NEBNext Ultra II (200 ng)	2,891,641,362	2,879,535,806	99.58	2,863,935,742	99.46	75.53	92.64	245.11	92.93	92.21	89.40	40.97	236

Libraries were made from NEBNext Ultra II (200 ng) library protocol and sequenced on Illumina HiSeq 4000.

**Online-Only Table 3:** Whole Exome Sequencing (WES) data sets for fresh DNA extracted from HCC1395BL and HCC1395 cell lines.

Sample ID	Sample	Biosample	Platform	Machine model	library prep protocol	Total Reads	Total Reads After Trimming	Percent Total Reads after Trimming	Total Mapped Reads	Percent Total Mapped Reads (Trimmed)	Percent Non-duplicated Reads (Mapped Trimmed)	Percent Reads Mapped On Target	Mean Coverage Inside Target (X)	Percent Coverage > = 15x	Percent Coverage > = 30x	Percent Coverage > = 100x	Percent GC	Median Insert Size
WES_IL_N_1	HCC1395BL	fresh cell	HiSeq	HiSeq 3000/4000	Agilent SureSelect Human All Exon v6 + UTRs	279,588,550	279,131,838	99.84	278,802,099	99.88	57.12	85.66	250	98.22	95.58	75.96	48.18	171
WES_IL_N_2	HCC1395BL	fresh cell	HiSeq	HiSeq 3000/4000	Agilent SureSelect Human All Exon v6 + UTRs	701,443,812	700,345,738	99.84	699,269,688	99.85	72.62	76.73	554	99.52	99.23	95.44	47.26	188
WES_IL_N_3	HCC1395BL	fresh cell	HiSeq	HiSeq 3000/4000	Agilent SureSelect Human All Exon v6 + UTRs	382,848,400	382,210,820	99.83	381,421,428	99.79	87.45	44.61	172	99.14	96.90	66.10	45.46	206
WES_EA_N_1	HCC1395BL	fresh cell	HiSeq	HiSeq 2500	Agilent SureSelect Human All Exon v6 + UTRs	176,997,814	175,449,424	99.13	175,389,529	99.97	71.88	86.94	165	96.47	92.23	61.91	50.28	161
WES_NC_N_1	HCC1395BL	fresh cell	HiSeq	HiSeq 2500	Agilent SureSelect Human All Exon v6 + UTRs	114,116,530	114,113,400	100.00	113,154,465	99.16	96.59	69.07	83	95.69	84.44	27.31	46.62	172
WES_LL_N_1	HCC1395BL	fresh cell	HiSeq	HiSeq 4000	Agilent SureSelect Human All Exon v6 + UTRs	69,810,764	69,803,466	99.99	69,388,404	99.41	75.04	76.08	89	87.00	78.31	14.15	49.57	174
WES_NV_N_1	HCC1395BL	fresh cell	HiSeq	HiSeq 2500	Agilent SureSelect Human All Exon v6 + UTRs	397,299,120	397,250,484	99.988	394,606,858	99.34	78.66	73.67	317	99.40	98.80	89.18	44.06	272
WES_NV_N_2	HCC1395BL	fresh cell	HiSeq	HiSeq 2500	Agilent SureSelect Human All Exon v6 + UTRs	418,058,694	418,034,480	99.994	414,260,963	99.10	79.46	75.1	333	99.42	98.84	89.69	43.76	271
WES_NV_N_3	HCC1395BL	fresh cell	HiSeq	HiSeq 2500	Agilent SureSelect Human All Exon v6 + UTRs	375,905,406	375,873,310	99.991	373,410,296	99.35	80.00	74.65	307	99.37	98.70	88.14	43.98	274
WES_FD_N_1	HCC1395BL	fresh cell	HiSeq	HiSeq 3000/4000	Agilent SureSelect Human All Exon v6 + UTRs	68,458,296	68,453,794	99.993	68,364,347	99.87	77.26	73.85	62	91.19	73.51	16.91	48.71	173
WES_FD_N_2	HCC1395BL	fresh cell	HiSeq	HiSeq 3000/4000	Agilent SureSelect Human All Exon v6 + UTRs	78,054,046	78,049,486	99.994	77,947,282	99.87	77.11	73.26	70	93.01	78.41	21.55	48.25	176
WES_FD_N_3	HCC1395BL	fresh cell	HiSeq	HiSeq 3000/4000	Agilent SureSelect Human All Exon v6 + UTRs	80,289,374	80,283,552	99.993	80,182,548	99.87	75.28	72.98	72	92.98	78.52	22.44	48.83	169
WES_IL_T_1	HCC1395	fresh cell	HiSeq	HiSeq 3000/4000	Agilent SureSelect Human All Exon v6 + UTRs	247,464,114	247,122,814	99.86	246,359,571	99.69	57.07	82.19	214	98.28	95.16	68.80	47.46	171
WES_IL_T_2	HCC1395	fresh cell	HiSeq	HiSeq 3000/4000	Agilent SureSelect Human All Exon v6 + UTRs	776,700,452	775,713,612	99.87	774,851,468	99.89	84.68	75.54	613	99.32	99.00	95.13	47.69	191
WES_IL_T_3	HCC1395	fresh cell	HiSeq	HiSeq 3000/4000	Agilent SureSelect Human All Exon v6 + UTRs	342,123,218	341,680,540	99.87	341,089,266	99.83	82.45	69.44	246	98.95	97.37	77.31	45.92	208
WES_EA_T_1	HCC1395	fresh cell	HiSeq	HiSeq 2500	Agilent SureSelect Human All Exon v6 + UTRs	210,071,430	208,191,476	99.11	208,119,731	99.97	77.36	86.72	197	95.89	92.14	65.97	49.55	165
WES_NC_T_1	HCC1395	fresh cell	HiSeq	HiSeq 2500	Agilent SureSelect Human All Exon v6 + UTRs	109,097,098	109,094,012	100.00	108,356,145	99.32	96.88	70.26	81	93.98	80.35	26.21	47.02	171
WES_LL_T_1	HCC1395	fresh cell	HiSeq	HiSeq 4000	Agilent SureSelect Human All Exon v6 + UTRs	53,498,898	53,493,162	99.99	53,302,502	99.64	74.53	58.44	89	78.21	66.11	7.94	50.12	174
WES_NV_T_1	HCC1395	fresh cell	HiSeq	HiSeq 2500	Agilent SureSelect Human All Exon v6 + UTRs	380,994,344	380,922,176	99.981	378,402,248	99.34	78.76	73.91	307	99.13	98.30	85.63	44.57	261
WES_NV_T_2	HCC1395	fresh cell	HiSeq	HiSeq 2500	Agilent SureSelect Human All Exon v6 + UTRs	398,077,794	398,060,634	99.996	394,293,828	99.05	80.50	73.22	311	99.15	98.34	85.89	44.4	278
WES_NV_T_3	HCC1395	fresh cell	HiSeq	HiSeq 2500	Agilent SureSelect Human All Exon v6 + UTRs	386,958,118	386,882,840	99.981	384,363,231	99.35	81.70	73.86	310	99.13	98.30	85.78	44.52	274
WES_FD_T_1	HCC1395	fresh cell	HiSeq	HiSeq 3000/4000	Agilent SureSelect Human All Exon v6 + UTRs	72,787,240	72,783,222	99.994	72,699,310	99.89	77.54	74.4	67	89.90	72.02	19.35	49.15	168
WES_FD_T_2	HCC1395	fresh cell	HiSeq	HiSeq 3000/4000	Agilent SureSelect Human All Exon v6 + UTRs	68,338,828	68,334,984	99.994	68,153,419	99.73	78.14	73.61	60	88.39	68.41	16.02	48.87	172
WES_FD_T_3	HCC1395	fresh cell	HiSeq	HiSeq 3000/4000	Agilent SureSelect Human All Exon v6 + UTRs	68,729,270	68,724,882	99.994	68,625,572	99.86	78.80	72.85	61	88.77	69.10	16.50	48.97	172

Libraries were made by using Agilent SureSelect Human All Exon v6 + UTRs library protocol and sequenced by six sequencing centers on Illumina HiSeq 2500 and HiSeq 3000/4000 for cross-site comparision.

**Online-Only Table 4:** Whole Exome Sequencing (WES) data sets for DNA extracted from HCC1395BL and HCC1395 cell lines and processed via Formalin-Fixed Paraffin-Embedded (FFPE) process with fixation time of 1, 2, 6, or 24 hours.

Sample ID	Sample	Biosample	Platform	Machine model	library Protocol	Total Reads (PF)	Total Reads After Trimming	Percent Total Reads after Trimming	Total Mapped Reads (Trimmed)	Percent Total Mapped Reads (Trimmed)	Percent Non-duplicated Reads (Mapped Trimmed)	Percent Reads Mapped On Target	Effect Mean Coverage Inside Target (X)	Percent Coverage > = 15x	Percent Coverage > = 30x	Percent Coverage > = 100x	Percent GC	Median Insert Size
FFX_GZ_T_1h_1	HCC1395	FFPE	HiSeq	HiSeq 4000	Agilent SureSelect Human All Exon v6 + UTRs	72,688,712	72,595,030	99.87	72,386,458	99.71	87.95	89.23	71	61.71	48.90	20.99	58.17	174
FFX_GZ_T_1h_3	HCC1395	FFPE	HiSeq	HiSeq 4000	Agilent SureSelect Human All Exon v6 + UTRs	104,142,794	103,976,012	99.84	103,701,635	99.74	86.35	83.89	96	68.06	63.55	35.76	53.83	173
FFX_GZ_T_6h_3	HCC1395	FFPE	HiSeq	HiSeq 4000	Agilent SureSelect Human All Exon v6 + UTRs	83,420,206	83,313,508	99.87	83,083,057	99.72	86.73	85.97	79	67.01	60.58	28.64	53.86	173
FFX_GZ_T_24h_1	HCC1395	FFPE	HiSeq	HiSeq 4000	Agilent SureSelect Human All Exon v6 + UTRs	126,758,774	126,608,628	99.88	126,157,724	99.64	86.25	88.59	122	68.14	62.15	34.19	57.67	178
FFX_GZ_T_24h_2	HCC1395	FFPE	HiSeq	HiSeq 4000	Agilent SureSelect Human All Exon v6 + UTRs	80,904,232	80,702,760	99.75	80,484,694	99.73	85.48	86.80	78	66.74	59.77	27.12	54.75	177
FFX_IL_T_1h_1	HCC1395	FFPE	HiSeq	HiSeq 4000	Agilent SureSelect Human All Exon v6 + UTRs	608,995,390	608,349,240	99.89	607,607,604	99.88	46.30	83.67	569	99.21	98.64	91.11	44.05	191
FFX_IL_T_2h_1	HCC1395	FFPE	HiSeq	HiSeq 4000	Agilent SureSelect Human All Exon v6 + UTRs	778,975,090	777,139,604	99.76	775,811,184	99.83	33.23	76.22	685	96.75	94.16	81.39	42.82	147
FFX_IL_T_6h_1	HCC1395	FFPE	HiSeq	HiSeq 4000	Agilent SureSelect Human All Exon v6 + UTRs	419,922,488	417,286,480	99.37	416,806,114	99.89	65.04	68.94	310	98.94	97.25	78.89	44.21	204
FFX_IL_T_24h_1	HCC1395	FFPE	HiSeq	HiSeq 4000	Agilent SureSelect Human All Exon v6 + UTRs	878,303,836	872,276,568	99.31	871,782,275	99.94	47.68	64.61	644	98.42	97.19	88.47	44.00	168
FFX_GZ_N_1h_1	HCC1395BL	FFPE	HiSeq	HiSeq 4000	Agilent SureSelect Human All Exon v6 + UTRs	110,247,342	109,510,666	99.33	109,202,128	99.72	85.61	87.30	105	68.78	64.80	39.58	53.89	178
FFX_GZ_N_2h_3	HCC1395BL	FFPE	HiSeq	HiSeq 4000	Agilent SureSelect Human All Exon v6 + UTRs	97,716,616	97,226,646	99.50	96,965,642	99.73	87.49	87.88	94	68.50	64.46	36.05	54.31	174
FFX_GZ_N_6h_1	HCC1395BL	FFPE	HiSeq	HiSeq 4000	Agilent SureSelect Human All Exon v6 + UTRs	96,772,432	95,979,612	99.18	95,684,076	99.69	85.75	88.64	92	67.53	60.93	28.26	57.13	172
FFX_GZ_N_24h_1	HCC1395BL	FFPE	HiSeq	HiSeq 4000	Agilent SureSelect Human All Exon v6 + UTRs	90,151,054	90,056,824	99.90	89,810,047	99.73	87.25	87.86	88	67.16	59.81	27.36	57.10	173
FFX_IL_N_1h_2	HCC1395BL	FFPE	HiSeq	HiSeq 4000	Agilent SureSelect Human All Exon v6 + UTRs	963,476,124	959,914,384	99.63	957,187,812	99.72	50.50	73.14	775	99.60	99.48	97.58	46.59	194
FFX_IL_N_2h_2	HCC1395BL	FFPE	HiSeq	HiSeq 4000	Agilent SureSelect Human All Exon v6 + UTRs	535,913,728	533,170,624	99.49	532,149,369	99.81	23.80	75.59	434	99.00	97.42	81.56	55.67	202
FFX_IL_N_6h_2	HCC1395BL	FFPE	HiSeq	HiSeq 4000	Agilent SureSelect Human All Exon v6 + UTRs	1,386,577,130	1,382,604,794	99.71	1,375,292,872	99.47	22.69	69.60	1058	99.64	99.56	98.55	46.66	186
FFX_IL_N_24h_2	HCC1395BL	FFPE	HiSeq	HiSeq 4000	Agilent SureSelect Human All Exon v6 + UTRs	1,041,349,526	1,040,887,252	99.96	1,039,335,968	99.85	25.82	81.78	942	99.63	99.52	97.93	44.65	206

Libraries were made from Agilent SureSelect Human All Exon v6 + UTRs protocol and sequenced on Illumina HiSeq 4000.

**Online-Only Table 5:** WGS Libraries were made from different library protocols such as TruSeq Nano, TruSeq PCR Free and Nextera Flex library protocol with different input amount and sequenced on Illumina HiSeq 3000/4000.

Sample ID	Sample	Biosample	Platform	Machine model	library prep protocol (Input amount)	Total Reads	Total Reads After Trimming	Percent Total Reads after Trimming	Total Mapped Reads	Percent Total Mapped Reads (Trimmed)	Percent Non-duplicated Reads	Mean Coverage Depth	Mean Coverage Depth SD	Percent of Coverage > = 5X	Percent GC	Median Insert Size
LBP_LL_T_1 ng	HCC1395	Fresh cells	HiSeq	HiSeq 4000	TruSeq-Nano (1ng)	926,143,044	892,177,246	96.3	887,751,031	99.5	17.4	32.3	128.4	88.2	40.3	411
LBP_LL_T_10 ng	HCC1395	Fresh cells	HiSeq	HiSeq 4000	TruSeq-Nano (10ng)	860,252,576	858,792,572	99.8	855,051,115	99.6	57.2	31.8	126.7	92.4	40.4	382
LBP_LL_T_100 ng	HCC1395	Fresh cells	HiSeq	HiSeq 4000	TruSeq-Nano (100ng)	840,067,566	834,660,190	99.4	830,420,093	99.5	83.4	30.7	127.7	92.6	40.3	384
LBP_LL_T_250 ng	HCC1395	Fresh cells	HiSeq	HiSeq 4000	TruSeq-PCR-free(250ng)	875,539,968	874,446,850	99.9	868,831,019	99.4	78.5	33.5	136.0	92.7	40.2	367
LBP_LL_N_1 ng	HCC1395BL	Fresh cells	HiSeq	HiSeq 4000	TruSeq-Nano (1ng)	863,716,968	857,386,678	99.3	852,997,339	99.5	26.4	31.6	169.3	92.3	40.1	382
LBP_LL_N_10 ng	HCC1395BL	Fresh cells	HiSeq	HiSeq 4000	TruSeq-Nano (10ng)	854,239,270	835,851,330	97.8	831,617,019	99.5	72.3	30.4	158.0	93.4	40.2	399
LBP_LL_N_100 ng	HCC1395BL	Fresh cells	HiSeq	HiSeq 4000	TruSeq-Nano (100ng)	1,230,957,576	1,227,609,086	99.7	1,221,602,690	99.5	76.5	44.7	237.4	93.6	40.1	405
LBP_LL_N_250 ng	HCC1395BL	Fresh cells	HiSeq	HiSeq 4000	TruSeq-PCR-free(250ng)	842,036,782	840,807,462	99.9	835,177,981	99.3	81.0	31.7	177.1	93.4	40.0	371
LBP_HS_N_100 ng_1	HCC1395BL	Fresh cells	HiSeq	HiSeq 4000	Nextera flex (100ng)	1,582,185,218	1,581,324,668	99.95	1,575,553,437	99.64	87.64	51.98	291.6	93.6	40.7	371
LBP_HS_N_10 ng_1	HCC1395BL	Fresh cells	HiSeq	HiSeq 4000	Nextera flex (10ng)	2,057,279,420	2,054,948,200	99.89	2,047,669,492	99.65	82.95	70.20	325.3	93.7	40.4	328
LBP_HS_N_1 ng_1	HCC1395BL	Fresh cells	HiSeq	HiSeq 4000	Nextera flex (1ng)	1,431,611,946	1,428,647,758	99.79	1,424,363,120	99.70	74.36	48.02	257.7	93.6	40.7	307
LBP_HS_T_100 ng_1	HCC1395	Fresh cells	HiSeq	HiSeq 4000	Nextera flex (100ng)	1,723,259,958	1,722,198,188	99.94	1,716,524,152	99.67	84.09	57.79	238.8	93.0	41.0	364
LBP_HS_T_10 ng_1	HCC1395	Fresh cells	HiSeq	HiSeq 4000	Nextera flex (10ng)	2,083,680,112	2,082,045,850	99.92	2,076,564,864	99.74	79.18	72.35	303.1	93.0	40.6	336
LBP_HS_T_1 ng_1	HCC1395	Fresh cells	HiSeq	HiSeq 4000	Nextera flex (1ng)	1,794,810,974	1,792,451,636	99.87	1,788,984,708	99.81	52.23	63.46	259.0	93.0	40.9	341

**Online-Only Table 6:** WGS Libraries were made from pooling the HCC1395 and HCC1395BL cell lines with various ratios (3:1, 1:1, 1:4, 1:9 and 1:19) to create mixtures.

Sample ID	Sample(Mix ratio)	Biosample	Platform	Machine model	library Protocol(Iinput amount)	Total Reads	Total Reads After Trimming	Percent Total Reads after Trimming	Total Mapped Reads	Percent Total Mapped Reads (Trimmed)	Percent Non-duplicated Reads	Mean Coverage Depth	Mean Coverage Depth SD	Percent of Coverage > = 5X	Percent GC	Median Insert Size
SPP_GT_0-1_1	HCC1395:HCC1395BL (0:1)	Fresh cells	HiSeq	HiSeq 4000	TruSeq DNA PCR Free (1000 ng)	3,076,097,436	3,076,097,436	98.6	3,066,844,772	99.7	83.0	115.0	657.4	93.7	40.0	380
SPP_GT_0-1_2	HCC1395:HCC1395BL (0:1)	Fresh cells	HiSeq	HiSeq 4000	TruSeq DNA PCR Free (1000 ng)	3,001,832,062	3,001,832,062	99.9	2,993,554,635	99.7	86.1	112.0	627.8	93.7	40.0	391
SPP_GT_0-1_3	HCC1395:HCC1395BL (0:1)	Fresh cells	HiSeq	HiSeq 4000	TruSeq DNA PCR Free (1000 ng)	2,903,255,740	2,903,255,740	99.9	2,893,798,551	99.7	86.7	105.1	586.8	93.7	39.9	378
SPP_GT_1-0_1	HCC1395:HCC1395BL (1:0)	Fresh cells	HiSeq	HiSeq 4000	TruSeq DNA PCR Free (1000 ng)	3,077,848,110	3,077,848,110	98.9	3,069,341,612	99.7	76.4	117.9	475.3	93.1	40.2	368
SPP_GT_1-0_2	HCC1395:HCC1395BL (1:0)	Fresh cells	HiSeq	HiSeq 4000	TruSeq DNA PCR Free (1000 ng)	3,090,006,648	3,090,006,648	99.9	3,082,407,730	99.8	83.5	115.7	476.2	93.1	40.2	370
SPP_GT_1-0_3	HCC1395:HCC1395BL (1:0)	Fresh cells	HiSeq	HiSeq 4000	TruSeq DNA PCR Free (1000 ng)	2,962,991,404	2,962,991,404	99.9	2,954,244,873	99.7	87.8	107.5	448.9	93.1	40.1	376
SPP_GT_1-1_1	HCC1395:HCC1395BL (1:1)	Fresh cells	HiSeq	HiSeq 4000	TruSeq DNA PCR Free (1000 ng)	3,055,617,586	3,055,617,586	99.6	3,046,293,478	99.7	79.9	114.2	538.5	93.8	40.0	379
SPP_GT_1-1_2	HCC1395:HCC1395BL (1:1)	Fresh cells	HiSeq	HiSeq 4000	TruSeq DNA PCR Free (1000 ng)	2,972,612,956	2,972,612,956	100.0	2,963,334,376	99.7	78.4	107.8	532.6	93.8	39.8	383
SPP_GT_1-1_3	HCC1395:HCC1395BL (1:1)	Fresh cells	HiSeq	HiSeq 4000	TruSeq DNA PCR Free (1000 ng)	3,070,633,366	3,070,633,366	100.0	3,058,122,892	99.6	89.7	106.9	493.9	93.8	40.3	396
SPP_GT_1-4_1	HCC1395:HCC1395BL (1:4)	Fresh cells	HiSeq	HiSeq 4000	TruSeq DNA PCR Free (1000 ng)	3,031,054,048	3,031,054,048	97.5	3,018,769,715	99.6	74.4	114.9	609.9	93.8	40.0	369
SPP_GT_1-4_2	HCC1395:HCC1395BL (1:4)	Fresh cells	HiSeq	HiSeq 4000	TruSeq DNA PCR Free (1000 ng)	2,984,886,368	2,984,886,368	99.9	2,974,891,252	99.7	86.5	107.9	574.8	93.8	40.0	395
SPP_GT_1-4_3	HCC1395:HCC1395BL (1:4)	Fresh cells	HiSeq	HiSeq 4000	TruSeq DNA PCR Free (1000 ng)	3,076,330,144	3,076,330,144	98.5	3,065,365,108	99.6	70.8	111.9	576.8	93.8	40.1	392
SPP_GT_1-9_1	HCC1395:HCC1395BL (1:9)	Fresh cells	HiSeq	HiSeq 4000	TruSeq DNA PCR Free (1000 ng)	3,169,041,440	3,169,041,440	98.9	3,160,284,662	99.7	65.8	117.6	662.4	93.8	39.9	383
SPP_GT_1-9_2	HCC1395:HCC1395BL (1:9)	Fresh cells	HiSeq	HiSeq 4000	TruSeq DNA PCR Free (1000 ng)	3,001,203,286	3,001,203,286	99.9	2,993,060,766	99.7	84.9	109.1	605.5	93.8	39.9	384
SPP_GT_1-9_3	HCC1395:HCC1395BL (1:9)	Fresh cells	HiSeq	HiSeq 4000	TruSeq DNA PCR Free (1000 ng)	3,142,707,394	3,142,707,394	99.2	3,133,546,333	99.7	68.5	114.0	626.6	93.8	39.9	389
SPP_GT_1-19_1	HCC1395:HCC1395BL (1:19)	Fresh cells	HiSeq	HiSeq 4000	TruSeq DNA PCR Free (1000 ng)	3,113,584,442	3,113,584,442	98.3	3,104,340,674	99.7	63.0	115.4	663.9	93.7	39.8	381
SPP_GT_1-19_2	HCC1395:HCC1395BL (1:19)	Fresh cells	HiSeq	HiSeq 4000	TruSeq DNA PCR Free (1000 ng)	2,927,043,064	2,927,043,064	99.9	2,917,934,331	99.7	88.5	107.8	594.4	93.7	39.9	395
SPP_GT_1-19_3	HCC1395:HCC1395BL (1:19)	Fresh cells	HiSeq	HiSeq 4000	TruSeq DNA PCR Free (1000 ng)	3,044,009,076	3,044,009,076	99.9	3,033,242,220	99.6	90.6	109.1	579.2	93.8	40.2	397
SPP_GT_3-1_1	HCC1395:HCC1395BL (3:1)	Fresh cells	HiSeq	HiSeq 4000	TruSeq DNA PCR Free (1000 ng)	3,065,804,606	3,065,804,606	99.5	3,056,714,849	99.7	79.3	117.3	509.2	93.8	40.1	363
SPP_GT_3-1_2	HCC1395:HCC1395BL (3:1)	Fresh cells	HiSeq	HiSeq 4000	TruSeq DNA PCR Free (1000 ng)	3,052,239,564	3,052,239,564	100.0	3,042,838,917	99.7	66.1	112.7	523.1	93.8	39.8	376
SPP_GT_3-1_3	HCC1395:HCC1395BL (3:1)	Fresh cells	HiSeq	HiSeq 4000	TruSeq DNA PCR Free (1000 ng)	2,975,199,024	2,975,199,024	99.9	2,966,022,353	99.7	86.4	108.0	481.4	93.8	40.0	375

Libraries were prepared by using TruSeq DNA PCR Free (1000 ng) protocol and sequenced on Illumina HiSeq 4000.

**Online-Only Table 7:** 10X Genomics Chromium Genome Sequencing (10X WGS) data sets for fresh DNA extracted from HCC1395BL and HCC1395 cell lines.

Sample ID	Sample	Biosample	Platform	Machine model	library Protocol (Input amount)	Total Reads	Total Mapped Reads	molecule length mean (Kb)	%mapped reads	mean depth	zero coverage	% pcr duplication	large sv calls	short deletion calls	longest phase block (Mb)	n50 phase block (Mb)
CHR_IL_T_1	HCC1395	Fresh cells	HiSeq	HiSeq 3000/4000	10X Chromium Genome Library preparation v2 (1250 ng)	2,212,061,014	2,092,851,168	72.00	94.61	92	1.42	8.32	336	4033	32	0.9
CHR_IL_T_2	HCC1395	Fresh cells	HiSeq	HiSeq 3000/4000	10X Chromium Genome Library preparation v2 (1250 ng)	2,266,945,480	2,147,917,753	76.00	94.75	94	1.41	7.03	338	4257	31.9	0.9
CHR_IL_T_3	HCC1395	Fresh cells	HiSeq	HiSeq 3000/4000	10X Chromium Genome Library preparation v2 (1250 ng)	2,329,482,706	2,226,020,206	77.00	95.56	99	1.40	6.44	330	4626	26.7	0.8
CHR_NC_T_1	HCC1395	Fresh cells	HiSeq	HiSeq 3000/4000	10X Chromium Genome Library preparation v2 (1250 ng)	1,406,971,948	1,366,399,528	54.00	97.12	61	1.53	5.84	304	4438	14.9	1.2
CHR_EA_T_1	HCC1395	Fresh cells	HiSeq	HiSeq X10	10X Chromium Genome Library preparation v2 (1250 ng)	1,321,617,070	1,279,515,774	67.00	96.81	57	1.40	11.87	295	5267	16.9	0.6
CHR_FD_T_1	HCC1395	Fresh cells	HiSeq	HiSeq X10	10X Chromium Genome Library preparation v2 (1250 ng)	1,064,469,438	1,015,823,822	64.00	95.43	45	1.45	5.63	266	4439	17.5	0.7
CHR_FD_T_2	HCC1395	Fresh cells	HiSeq	HiSeq X10	10X Chromium Genome Library preparation v2 (1250 ng)	1,070,778,002	1,027,173,140	66.00	95.93	45	1.45	4.37	265	4313	15.2	0.7
CHR_FD_T_3	HCC1395	Fresh cells	HiSeq	HiSeq X10	10X Chromium Genome Library preparation v2 (1250 ng)	1,070,863,374	1,020,779,776	66.00	95.32	45	1.50	7.10	258	4382	18.4	0.8
CHR_NV_T_1	HCC1395	Fresh cells	HiSeq	HiSeq 4000	10X Chromium Genome Library preparation v2 (1250 ng)	2,763,309,906	2,686,525,126	67.60	97.22	119	1.44	12.05	353	3863	22.9	0.7
CHR_NV_T_2	HCC1395	Fresh cells	HiSeq	HiSeq 4000	10X Chromium Genome Library preparation v2 (1250 ng)	2,604,341,620	2,529,765,476	62.77	97.14	113	1.43	14.52	354	3705	17.6	0.6
CHR_NV_T_3	HCC1395	Fresh cells	HiSeq	HiSeq 4000	10X Chromium Genome Library preparation v2 (1250 ng)	2,614,247,622	2,546,038,714	61.83	97.39	114	1.43	13.23	350	3701	20.0	0.6
CHR_IL_N_1	HCC1395BL	Fresh cells	HiSeq	HiSeq 3000/4000	10X Chromium Genome Library preparation v2 (1250 ng)	2,134,749,498	2,025,432,377	70.00	94.88	90	0.82	7.50	25	3507	33.6	7.5
CHR_IL_N_2	HCC1395BL	Fresh cells	HiSeq	HiSeq 3000/4000	10X Chromium Genome Library preparation v2 (1250 ng)	2,202,527,584	2,107,633,315	72.00	95.69	93	0.82	7.16	23	3532	30.3	6.7
CHR_IL_N_3	HCC1395BL	Fresh cells	HiSeq	HiSeq 3000/4000	10X Chromium Genome Library preparation v2 (1250 ng)	2,380,468,964	2,279,808,496	70.00	95.77	101	0.81	6.98	24	3460	24.4	7
CHR_NC_N_1	HCC1395BL	Fresh cells	HiSeq	HiSeq 3000/4000	10X Chromium Genome Library preparation v2 (1250 ng)	1,420,063,110	1,383,151,777	66.00	97.40	62	0.90	6.79	24	3751	19.8	4.5
CHR_EA_N_1	HCC1395BL	Fresh cells	HiSeq	HiSeq X10	10X Chromium Genome Library preparation v2 (1250 ng)	1,204,338,932	1,157,467,982	67.00	96.11	52	0.87	10.70	21	4717	19.3	4.4
CHR_FD_N_1	HCC1395BL	Fresh cells	HiSeq	HiSeq X10	10X Chromium Genome Library preparation v2 (1250 ng)	1,079,294,448	1,047,436,008	63.00	97.05	46	0.91	5.02	24	3824	27.8	3.8
CHR_FD_N_2	HCC1395BL	Fresh cells	HiSeq	HiSeq X10	10X Chromium Genome Library preparation v2 (1250 ng)	1,074,129,460	1,037,897,214	63.00	96.63	46	0.92	4.47	20	3827	18.4	3.9
CHR_FD_N_3	HCC1395BL	Fresh cells	HiSeq	HiSeq X10	10X Chromium Genome Library preparation v2 (1250 ng)	1,069,034,706	1,026,145,348	61.00	95.99	45	0.79	4.65	20	3994	15.7	3.3
CHR_NV_N_1	HCC1395BL	Fresh cells	HiSeq	HiSeq 4000	10X Chromium Genome Library preparation v2 (1250 ng)	2,745,515,070	2,673,917,830	61.23	97.39	119	0.83	10.72	85	3063	25.4	4.7
CHR_NV_N_2	HCC1395BL	Fresh cells	HiSeq	HiSeq 4000	10X Chromium Genome Library preparation v2 (1250 ng)	2,610,495,528	2,542,805,354	55.45	97.41	114	0.83	14.18	93	3165	30.0	4.2
CHR_NV_N_3	HCC1395BL	Fresh cells	HiSeq	HiSeq 4000	10X Chromium Genome Library preparation v2 (1250 ng)	2,614,793,942	2,538,150,020	56.52	97.07	113	0.83	12.25	93	3181	30.3	4.4

Libraries were made from Chromium Genome Library preparation v2 kit and sequenced by five sequencing centers on Illumina HiSeq 3000/4000, HiSeq X10 for cross-site comparision.

**Online-Only Table 8:** PacBio Sequel II Whole Genome Sequencing data sets for fresh DNA extracted from HCC1395BL and HCC1395 cell lines.

Sample ID	Sample	Biosample	Platform	Machine model	library Protocol (Input amount)	Total Reads	Total Bases (Bps)	Total Mapped Reads	Percent Total Mapped Reads	Percent of PCR duplidate(mapped reads)	Mean Coverage Depth	Mean Coverage Depth SD	Percent of Coverage > = 5X	Percent GC	Median Read Lengh	Raw Coverage
PBO_Normal	HCC1395BL	Fresh cells	PacBio_Sequel	PacBio_Sequel II	Preparing >30 kbp SMRTbell Libraries Using Megarupter Shearing and Blue Pippin Size-Selection for PacBio RS II and Sequel Systems	21,935,561	131,842,758,047	16,759,622	76.40	2.46	44.17	33.25	92.29	41.14	8,050	44
PBO_Tumor	HCC1395	Fresh cells	PacBio_Sequel	PacBio_Sequel II	Preparing >30 kbp SMRTbell Libraries Using Megarupter Shearing and Blue Pippin Size-Selection for PacBio RS II and Sequel Systems	18,835,934	116,009,882,995	16,273,225	86.39	2.58	38.95	47.35	91.62	41.57	8,050	39

Libraries were made from PacBio library preparation and sequencing library protocol.

**Online-Only Table 9:** AmpliSeq libraries were prepared using Illumina protocol and sequenced on MiSeq platform.

Sample ID	Sample	Biosample	Platform	Machine model	library Protocol (Input amount)	Total Reads	Percent Total Reads after Trimming	Total Mapped Reads (Trimmed)	Percent Total Mapped Reads (Trimmed)	Percent Non-duplicated Reads (Mapped Trimmed)	Percent Reads Mapped On Target	Mean Coverage Inside Target (X)	Percent Coverage > = 30x	Percent GC	Median Insert Size
AMS_AB_N_1	HCC1395BL	Fresh cells	MiSeq	MiSeq	AmpliSeq	7,569,042	99.95	7,548,971	99.79	4.68	67.55	1173.88	98.61	41.66	191
AMS_AB_N_2	HCC1395BL	Fresh cells	MiSeq	MiSeq	AmpliSeq	6,801,804	99.97	6,783,534	99.76	4.45	66.44	1049.06	98.52	41.02	196
AMS_AB_N_3	HCC1395BL	Fresh cells	MiSeq	MiSeq	AmpliSeq	7,335,112	99.97	7,316,766	99.78	4.38	67.42	1155.25	98.52	40.95	196
AMS_AB_T_1	HCC1395	Fresh cells	MiSeq	MiSeq	AmpliSeq	8,245,680	99.96	8,225,203	99.79	4.34	66.10	1277.06	96.69	41.35	196
AMS_AB_T_2	HCC1395	Fresh cells	MiSeq	MiSeq	AmpliSeq	8,011,520	99.96	7,989,728	99.77	4.38	65.63	1238.92	96.71	41.46	196
AMS_AB_T_3	HCC1395	Fresh cells	MiSeq	MiSeq	AmpliSeq	9,163,270	99.96	9,141,920	99.81	4.33	65.89	1430.02	96.80	41.28	196

**Online-Only Table 10:** Single cell libraries were prepared using 10X Genomics Chromium Single Cell CNV Solution for CNV profiling.

Sample	total num reads	percent bases R1 Q30	percent bases R2 Q30	correct bc rate	percent non cell barcode	shortest primary contig	percent mappable bins	num cells	total num reads in cells	total num mapped dedup reads in cells	median percent mapped duplicates per cell	mean mapped dedup reads per cell	median effective reads per 1Mbp	median unmapped frac	mean ploidy p25	mean ploidy p50	mean ploidy p75	raw mapd p25	raw mapd p50	raw mapd p75	normalized mapd p25	normalized mapd p50	normalized mapd p75	normalized dimapd p25	normalized dimapd p50	normalized dimapd p75	raw dimapd p25	raw dimapd p50	raw dimapd p75	percent noisy cells	median est cnv resolution mb
TGEN_T1	1,260,880,054	0.93586572	0.84779037	0.87253147	0.03572204	46709983	0.90348588	1465	1060857656	870930774	0.05134489	594491.996	188	0.03222793	2.71181349	2.75221867	2.79193475	0.12499719	0.13813647	0.1566726	0.12499719	0.13813647	0.1566726	0.90367638	0.92666699	0.95082236	0.90367638	0.92666699	0.95082236	0.08395904	1.46801758
TGEN_N1	1,308,946,506	0.94857232	0.86500184	0.86586948	0.05646006	46709983	0.90344055	983	1069386312	817062608	0.12479393	831192.887	255	0.02080845	1.89378524	1.90302472	1.92504924	0.11509274	0.13183257	0.15403324	0.11509274	0.13183257	0.15403324	0.92951399	0.9629042	1.0362302	0.92951399	0.9629042	1.0362302	0.26347915	1.28393555
