# Supplementary material for: Whole genome and exome sequencing reference datasets from a multi-center and cross-platform benchmark study
Source: Sci Data. 2021 Nov 9;8:296. doi: 10.1038/s41597-021-01077-5 (PMC8578599; doi:10.1038/s41597-021-01077-5)
Supplement: Supplementary file 1 — Supplementary Figures [file 41597_2021_1077_MOESM1_ESM.pdf]

Whole genome and exome sequencing reference datasets from a multi-center and cross-platform benchmark study

Yongmei Zhao[1#], Li Tai Fang[2], Tsai-wei Shen[1], Sulbha Choudhari[1], Keyur Talsania[1], Xiongfong Chen[1], Jyoti Shetty[3], Yuliya Kriga[3], Bao Tran[3], Bin Zhu[4], Zhong Chen[5], Wanqiu Chen[5], Charles Wang[5], Erich Jaeger[6], Daoud Meerzaman[7], Charles Lu[8], Kenneth Idler[8], Luyao Ren[9], Yuanting Zheng[9], Leming Shi[9], Virginie Petitjean[10], Marc Sultan[10], Tiffany Hung[11], Eric Peters[11] Jiri Drabek[12,13], Petr Vojta[12,13], Roberta Maestro[13,14], Daniela Gasparotto[13,14], Sulev Kõks[13,15,16], Ene Reimann[13,17], Andreas Scherer[13,18], Jessica Nordlund[13,19], Ulrika Liljedahl[13,19], Jonathan Foox[20], Christopher E. Mason[20], Chunlin Xiao[21], Huixiao Hong[22], Wenming Xiao[23#]

TABLE OF CONTENTS

Supplementary Figures Page 2
Suppl. Figure 1: Preprocessing and QC analysis pipelines Page 2
Suppl. Figure 2: WGS and WES cross-site data quality metrics Page 3
Suppl. Figure 3: GC coverage bias Page 3
Suppl. Figure 4: Cumulative genome coverage for WGS and WES Page 4
Suppl. Figure 5: HCC1395 tumor genome ploidy and heterogeneity Page 5
Suppl. Figure 6: Source of DNA damage artifacts Page 6
Suppl. Figure 7: Mutation calling repeatability and O_Score distribution Page 7

Supplementary Figures

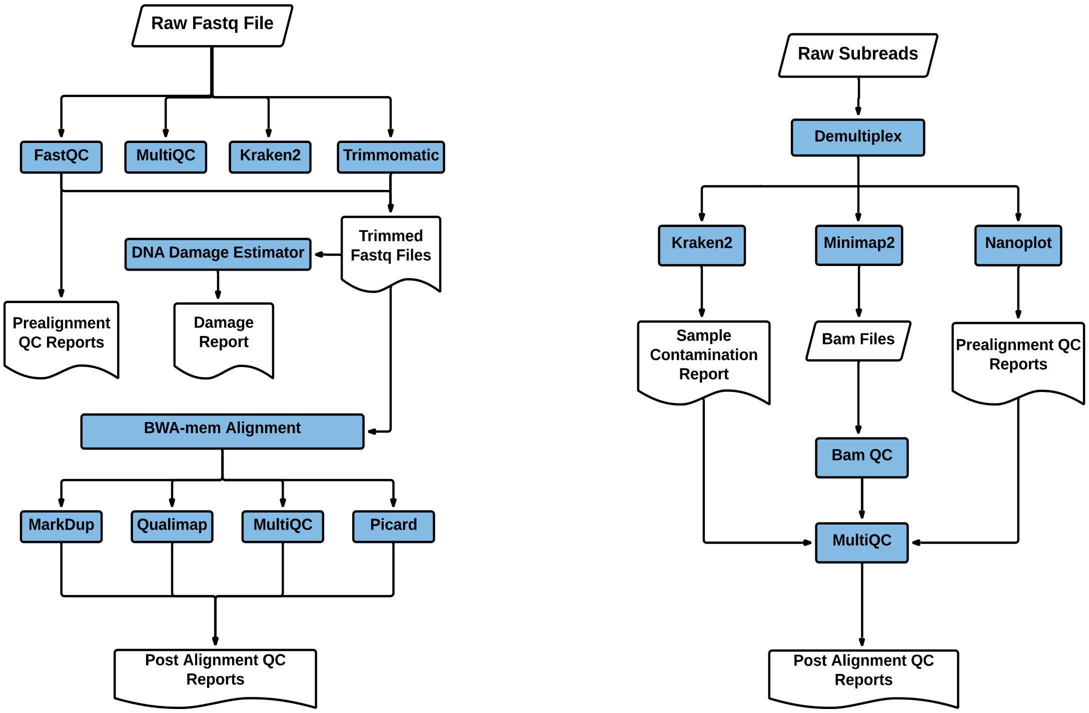

(a) (b)

Suppl. Figure 1: Preprocessing and QC analysis pipelines used for whole genome and whole exome-seq data analysis **(a)** Short-read Illumina sequencing QC pipeline. **(b)** PacBio long-read sequencing QC pipeline.

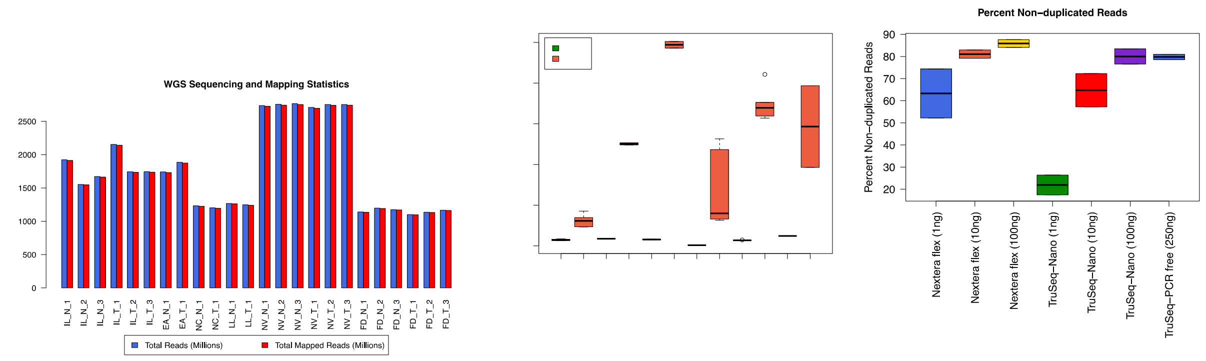

(a) (b) (c)

Suppl. Figure 2: WGS and WES cross-site data quality metrics **(a)** WGS cross-site sequencing yields (Millions) mapped reads (Millions) statistics. **(b)** Adapter contents in WGS and WES Illumina short-read data set across 6 data centers. **(c)** None duplicate mapped reads in Nextera Flex, TruSeq Nano and TruSeq PCR free protocols with different input amount**.**

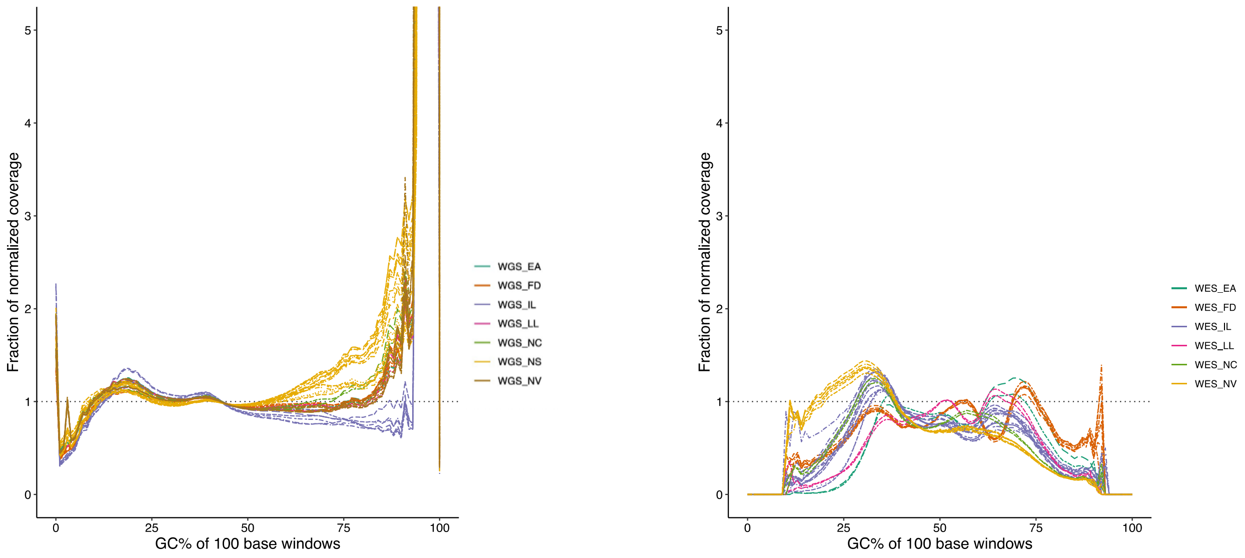

(a) (b)

Suppl. Figure 3: GC Coverage Bias - using the Picard CollectGCBiasMetrics to measure the relative GC against the sequencing coverage to show bias in coverage across regions of the genome with varying GC content. A perfect library would be a flat line at y = 1. The plot X-axis denotes the GC content bin from the corresponding reference sequence ranging from 0 - 100%. Y-axis denotes the normalized coverage measurement of sequence depth for the particular GC bin for WGS data **(a)** and WES data **(b).**

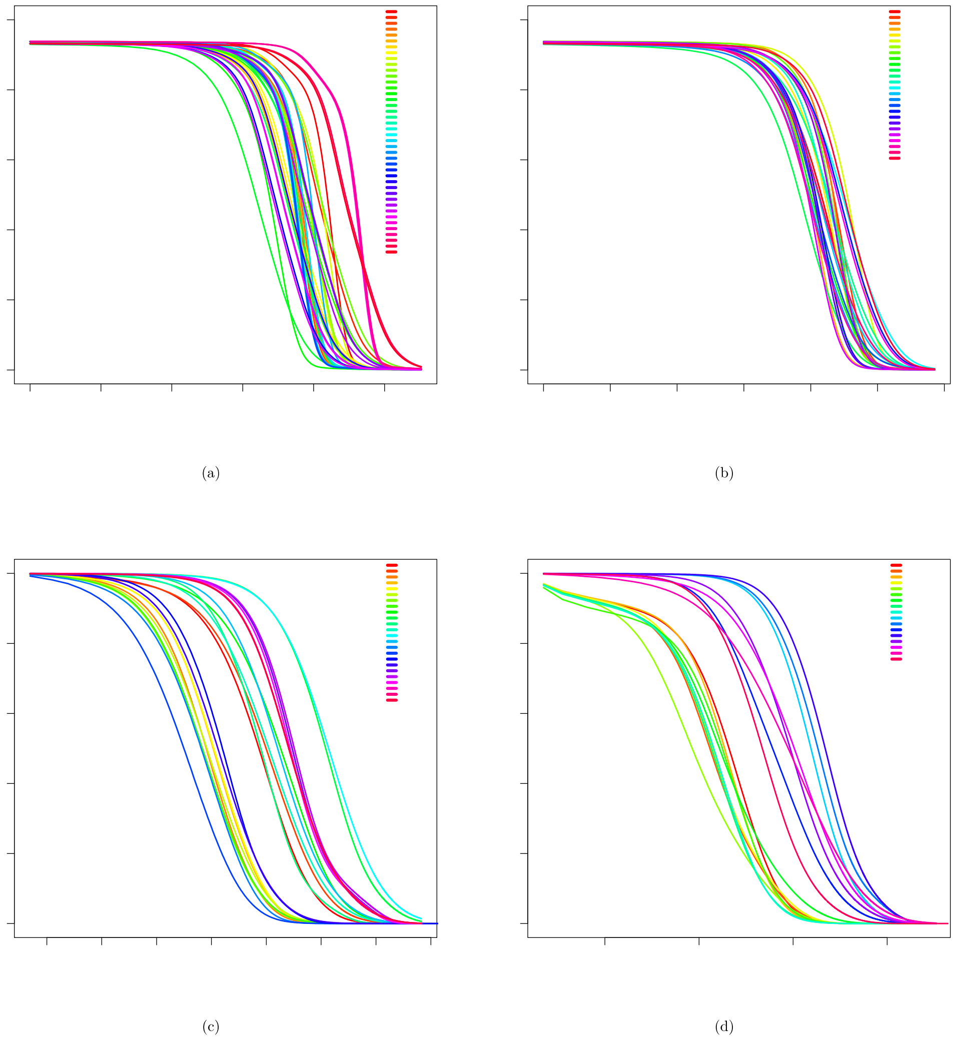

Suppl. Figure 4: Cumulative genome coverage for WGS and WES cross-site data sets and FFPE WGS and FFPE WES data sets. The graph displays the percentages of the reference genome with at least the given depth of coverage in log scale for each sample **(a)** Genome coverage for each fresh DNA prepared Illumina WGS libraries for cross-site comparison. **(b)** Genome coverage for FFPE WGS libraries. **(c)** Genome coverage for each Fresh DNA prepared Agilent SureSelect V6+UTR exome capture libraries for cross-site comparison. **(d)** Genome coverage for FFPE whole exome capture libraries.

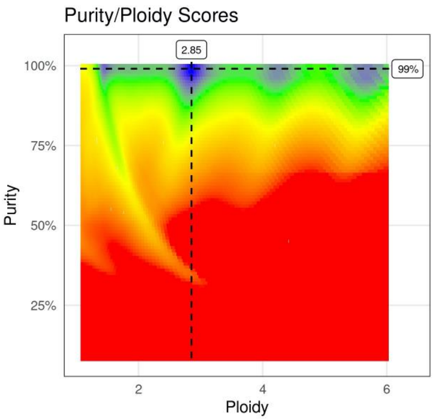

(a)

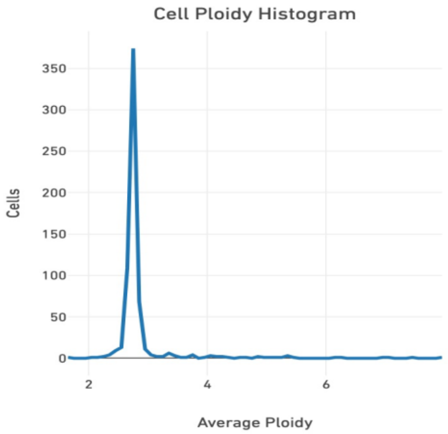

(b)

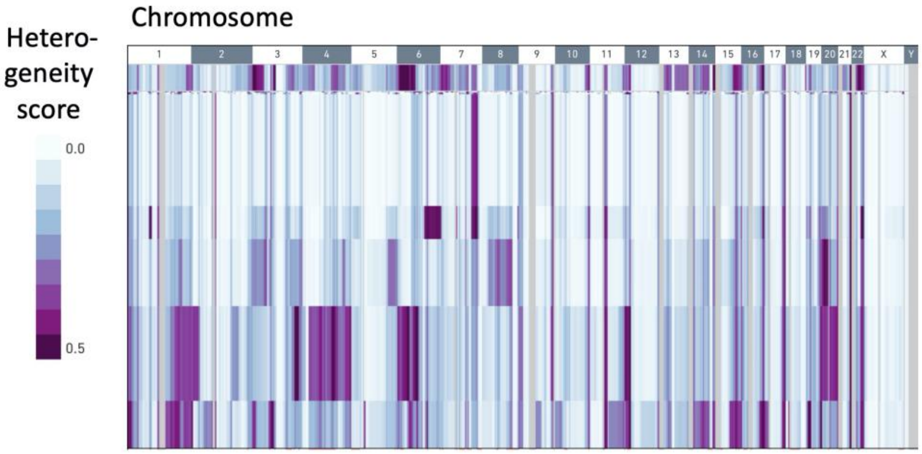

(c)

Suppl. Figure 5: HCC1395 tumor genome ploidy and heterogeneity measured from whole genome Illumina sequencing data and single cell CNV data. **(a)** Sample purity and ploidy estimated based on WGS for HCC1395 from Purple software, the tumor purity is above 99% with ploidy of 2.85. **(b)** Using 10X Genomics Single Cell CNV Solution, based on the analysis of 1270 cells for HCC1395 from 10x Single Cell CNV data set, Cellranger ploidy histogram displayed the vast majority of cells have ploidy of 2.8 as shown. **(c)** Heterogeneity analysis from10x Single Cell CNV data. Each row represents a cell being sequenced. Integer-scaled CNA profiles across the genome of 1270 HCC1395 cells were obtained. Similar cells were clustered together based on CNAs. Subclonal populations are marked in tracks. The chromosome-scale gains showed in darker purple, and losses displayed as light color in heatmap.

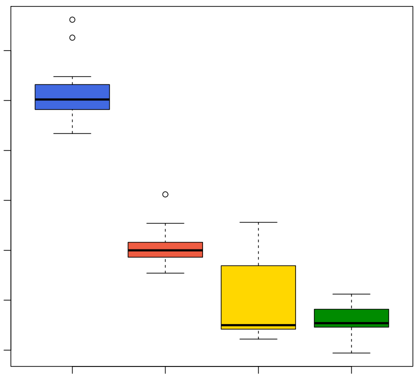

(a)

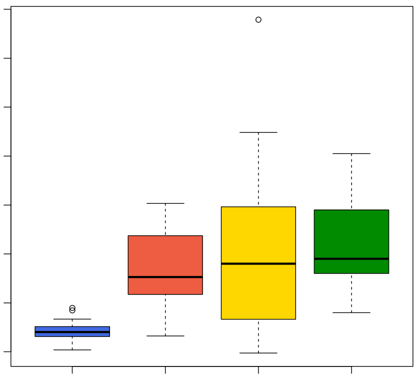

(b)

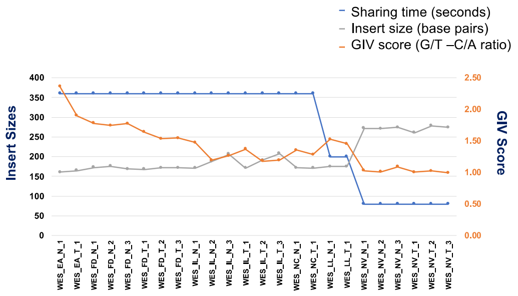

(c)

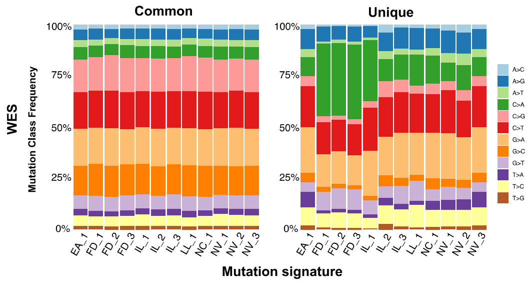

(d)

Suppl. Figure 6: Source of DNA damage artifacts and effect on cancer genome mutation calls. One type of DNA damage artifacts is introduced during DNA fragment mechanical shearing. Longer library inserts shave lower DNA damage scores (GIV scores) as shown in **(a)** library insert sizes for WGS cross-site libraries, WGS FFPE libraries, WES cross-site libraries, and WES FFPE libraries. **(b)** GIV score across different data sets; **(c)** correlation between library shearing time, insert sizes, and GIV scores. **(d)** percentage of mutation types for WES. The shared mutation across replicates is shown in left plot or sample specific unique mutations are shown in right plot. Note, high percentage of C/A mutation in the sites that also have high G/T_C/A GIV scores as displayed in **Suppl. Figure 6c**.

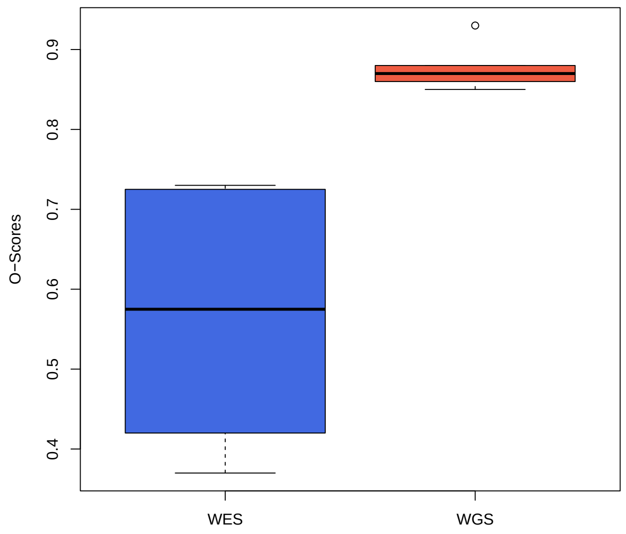

Suppl. Figure 7: Mutation calling repeatability and O_Score distribution for 12 WES and WGS runs. One-tailed t-tests of WES and WGS two groups has P-value of 3.32354E-07.
